# Global and regional erosion of mammalian functional diversity across the diel cycle

**DOI:** 10.1126/sciadv.abn6008

**Published:** 2022-08-12

**Authors:** Daniel T. C. Cox, Alexandra S. Gardner, Kevin J. Gaston

**Affiliations:** Environment and Sustainability Institute, University of Exeter, Penryn, Cornwall TR10 9FE, UK.

## Abstract

Biodiversity is declining worldwide. When species are physically active (i.e., their diel niche) may influence their risk of becoming functionally extinct. It may also affect how species losses affect ecosystems. For 5033 terrestrial mammals, we predict future changes to diel global and local functional diversity through a gradient of progressive functional extinction scenarios of threatened species. Across scenarios, diurnal species were at greater risk of becoming functionally extinct than nocturnal, crepuscular, and cathemeral species, resulting in deep functional losses in global diurnal trait space. Redundancy (species with similar roles) will buffer global nocturnal functional diversity; however, across the land surface, losses will mostly occur among functionally dispersed species (species with distinct roles). Functional extinctions will constrict boundaries of cathemeral trait space as megaherbivores, and arboreal foragers are lost. Variation in the erosion of functional diversity across the daily cycle will likely profoundly affect the partitioning of ecosystem functioning between night and day.

## Teaser

Effects of human pressures on global and regional mammalian functional diversity depend on when species are active.

## INTRODUCTION

The world has entered its sixth mass extinction (*1*–*3*). The current loss of vertebrate species is estimated to be ~1000 times faster than the background rate of extinctions from the fossil record (*4*). However, although species extinctions are important in the long run, focusing on these ignores the serious declines in abundance in extant populations that may be ~10 times more frequent and are a more immediate threat to ecosystems (*2*). These declines may often result in species no longer being sufficiently abundant to maintain critical interactions that ensure functionality and stability of ecological communities; functional extinction may occur even before losing 30% of their individuals (*5*).

When an animal is physically active (i.e., its diel niche) may be an important, but largely unexplored, dimension of functional extinction. There are three main reasons. First, anthropogenic pressures on the environment are not applied equally across the diel cycle (in part as a consequence of timings of human activity) and thus can differentially affect species that are active at particular times of day. In particular, climate change, pollution (e.g., atmospheric, sound, and light), harvesting, and disturbance all regularly exhibit strong diel variation [e.g., (*6*–*9*)]. For example, nighttime temperatures are warming faster than those during the day across much of the land surface, with impacts on carbon cycling, primary production, vegetation dynamics, and other ecosystem processes (*6*, *10*). Harvesting and disturbance pressures, on the other hand, may be greater during the daytime (*7*, *8*). Along with unsustainable levels of mortality disrupting trophic cascades and ecosystem functioning [e.g., (*11*, *12*)], these pressures are driving increasing numbers of species to switch the timing of their activity to the nighttime (*7*), further changing the diel community structure. It is likely that the negative impacts of many anthropogenic pressures on species are additive or synergistic, further increasing the impacts on species active during certain periods of the day (*13*–*15*).

Second, a species’ diel niche may be a driver of the evolution of its functional traits (*16*, *17*). Given that certain combinations of traits increase a species’ vulnerability to human pressures on the environment (*18*–*20*), this, in turn, means that species that are physically active during certain periods of the day may inherently be at greater risk than those active during other periods.

Third, the period during which an animal is physically active will determine when it primarily exerts its influence on ecosystems [e.g., predator-prey relationships (*21*), herbivory (*22*), and post-dispersal seed predation (*23*)]. This means that the loss of species from one diel niche may result in the loss or diminishment of the ecosystem functions they provide if species from other diel niches are unable to replace their functions or are unable to do these functions as effectively (i.e., there is limited functional redundancy across diel niches). For example, pollination by bats may not be carried out as effectively by their diurnal counterparts (*24*), while nocturnal mammals often disperse seeds at greater distances than diurnal birds do (*25*). Of particular concern here is the loss of functionally dispersed species (species that occupy unique regions of trait space) within diel niches, as these tend to have more marked consequences for ecosystem functioning (*26*, *27*).

Here, across 5033 extant terrestrial mammal species, we examine how functional extinctions would affect diel patterns of functional diversity by simulating a progressive loss of threatened species from functional trait space from the most to the least threatened. On the basis of the most recent International Union for Conservation of Nature (IUCN) Red List classifications (*28*), we first removed the most threatened species [critically endangered (CR)], before progressively removing additional species from lower categories of threat [CR + endangered (EN), CR + EN + vulnerable (VU), and CR + EN + VU + near threatened (NT) + threatened data deficient (DD)]. Under each functional extinction scenario, we determine (i) how the risk of functional extinction varies with when species are physically active; (ii) how functional extinctions will reorganize the boundaries and internal structure of global trait spectra of each diel niche [two-dimensional surfaces representing variation in species ecological strategies as defined by trait combinations (*16*, *29*–*32*)]; (iii) biogeographical patterns of functional extinctions in each diel niche; and (iv) whether biogeographical losses are occurring among functionally dispersed or functionally redundant species in each diel niche, relative to null expectations where species losses occur at random. Mammals provide an interesting study group: They exhibit a wide diversity of diel niches (*33*), have important ecological roles (*34*, *35*), and are highly threatened by human activities (*18*, *36*–*39*).

Across all functional extinction scenarios, we find an elevated risk of functional extinction in diurnal terrestrial mammals, whose activities more closely coincide with that of humans, compared to nocturnal, crepuscular, and cathemeral species. Within global trait space, high nocturnal functional redundancy will buffer functional diversity from the loss of threatened species; however, across much of the land surface, losses will occur among functionally dispersed species that inflates lost functional diversity compared to null expectations. The loss of threatened megafauna and arboreal foragers will constrict the boundaries of cathemeral trait space, resulting in the complete loss of trait combinations. The decimation of diurnal primates will result in deep functional losses in global trait space, and, geographically, this will manifest in the disproportionate loss of functionally dispersed species from across much of the tropics.

## RESULTS

### Global variation in functional extinctions across the diel cycle

Under all functional extinction scenarios, we found that diurnal mammals are at a greater risk of becoming functionally extinct than species occupying other diel niches (table S1); the most extreme scenario, where the functional influence of all currently threatened species is lost (IUCN status of CR, EN, VU, NT, or threatened DD), resulted in the loss of 41.3% of 896 diurnal species, 31.6% of 3498 nocturnal species, 34.8% of 113 crepuscular species, and 34.9% of 526 cathemeral species (table S1 and data files S1 and S2). This translates into an increased risk of functional extinction in diurnal compared to nocturnal species (estimate: 0.5 ± 0.1, *P* < 0.001), while there was no difference in the risk of functional extinction between crepuscular (estimate: 0.3 ± 0.3) or cathemeral (estimate: 0.2 ± 0.11) and nocturnal species (table S2).

### Shifting densities in diel global trait spectra

**Fig. 1.**
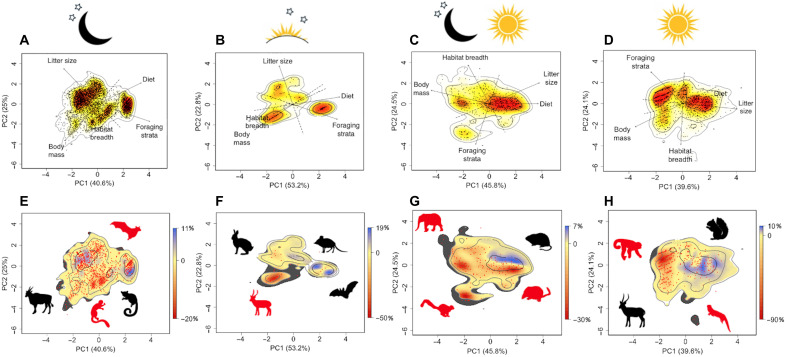
Erosion of global diel trait spectra following the NT functional extinction scenario. Namely, species classified as CR, EN, VU, NT, and threatened DD were removed. We give the global trait spectrum of all species for (**A**) nocturnal (moon and stars), (**B**) crepuscular (sunset/sunrise and stars), (**C**) cathemeral (moon, stars, and sun), and (**D**) diurnal mammals (sun). Projections show the species (dots) defined by PC axes, and percentage values give the proportion of the total variation explained. Solid arrows indicate the direction and weighting of traits analyzed. The color gradient specifies regions of the highest (red) to the lowest (white) occurrence probability of species, with contour lines indicating 0.5, 0.95, and 0.99 quantiles. (**E**) to (**H**) depict shifts in density of species occupation in the corresponding diel niche if threatened species are lost. Contour lines show quantiles for nonthreatened species, and red dots are the position of threatened species. Red reflects areas where estimated density is lower following functional extinctions (i.e., those traits become relatively less frequent at the global scale), and blue shows where estimated density is higher after extinctions (i.e., those traits become relatively more frequent at the global scale). Gray areas are functional spaces that are within the 0.99 quantile for all species but above the 0.99 quantile for nonthreatened species (i.e., a reduction in volume of trait space). Silhouettes represent nonthreatened (black) and threatened (red) species characterizing the corresponding regions of the trait spectra (downloaded from PhyloPic at www.phylopic.org).

We determined variation across diel niches in predicted changes of global trait space under progressive functional extinction scenarios. For each diel niche, in turn, we ordinated five functional traits (body mass, litter size, habitat breadth, foraging strata, and diet) to represent global trait spectra (two-dimensional surfaces mapping the position and density of species in trait space; Fig. 1, A to D) (*16*, *29*–*32*). Instead of simply characterizing the boundaries of trait space, this approach represents the functional spectra landscape with areas of high and low density of species occupation and allows the testing of changes in aspects of trait space occupation other than volume (*16*, *29*–*32*). Each trait spectrum contains hotspots—areas of particularly dense species occupation that, when combined, encompass 50% of species—that are unique to that trait spectrum (*31*). Global trait spectra varied across diel niches, with speciose Chiroptera driving similar patterns of species occupation in nocturnal and crepuscular species that, in turn, are different to those of cathemeral and diurnal trait spectra (Fig. 1, A to D) (*16*).

**Fig. 2.**
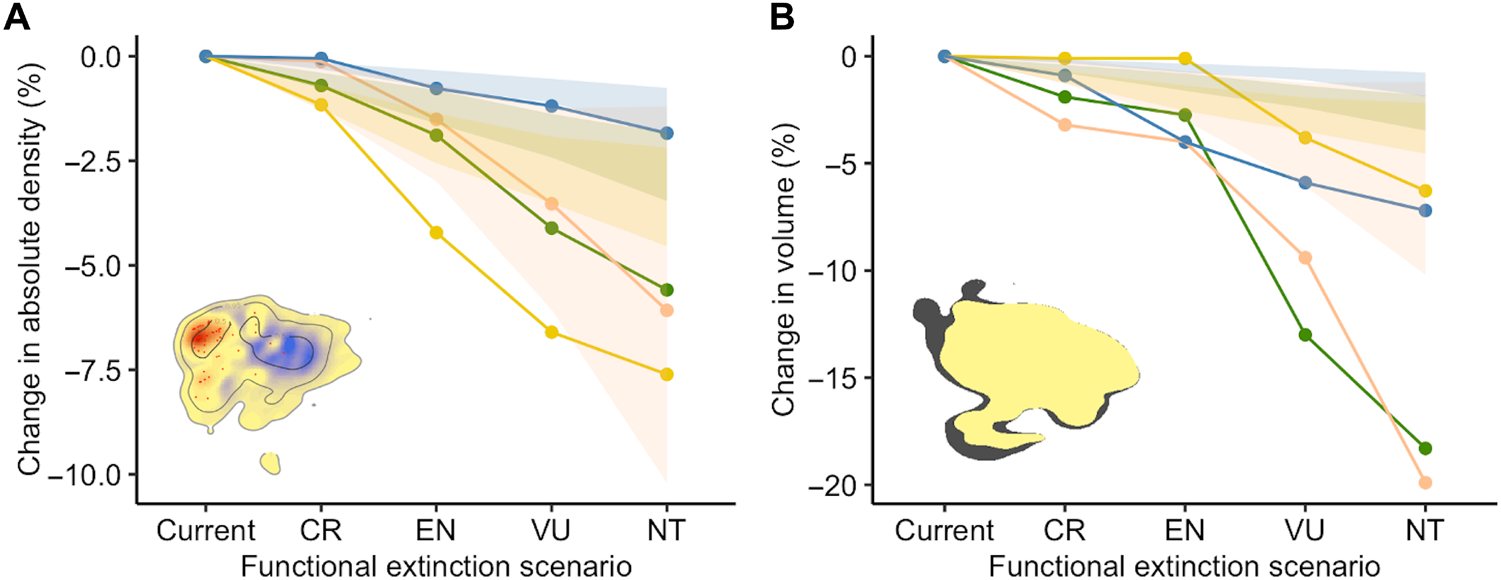
Loss of functional diversity of diel trait spectrum under progressive functional extinction scenarios. We estimated (**A**) the mean change in absolute density within the trait spectrum and (**B**) the change in volume. Points show the corresponding change in nocturnal (blue), crepuscular (peach), cathemeral (green), and diurnal (yellow) trait spectrum. We started by removing species with a higher risk of becoming functionally extinct (i.e., CR), then we continued progressively removing species from the categories with lower IUCN threat categories. In the figure, the name of the scenario refers to the lowest IUCN threat category simulated as functionally extinct. For each scenario, we compared the loss of each dimension of functional diversity with 100 repetitions of a null model where threatened species were randomly selected among all species in the diel niche. The 100 repetitions of the null model are represented as a polygon of the 95% confidence interval. Spectrum images on the panels illustrate examples of (A) change in density of species occupation and (B) change in volume.

Simulating the progressive functional loss of threatened species for each diel trait spectrum, we examined predicted changes in relative density of species occupation (i.e., species with similar combinations of traits) and changing boundaries (edges of occupied trait space) (*30*, *31*) as the influence of functionally redundant (i.e., with similar trait combinations) and functionally dispersed (i.e., relatively isolated in trait space) species is lost. Regardless of the extinction scenario, the deepest functional losses occur in diurnal trait space and the least occur in nocturnal trait space, while crepuscular and cathemeral mammals experience the greatest constriction to the boundaries of trait space (Figs. 1, E to H, and 2B; fig. S1; and table S3). As the extinction scenarios progress, functionally extinct species show distinct occupation patterns, evidenced by the low similarity in the relative density of species occupation between the trait spectra for nonthreatened species and the trait spectra for all threatened species (fig. S2).

Since functional richness (i.e., the volume of trait space occupied by species) is related to taxonomic richness (*40*, *41*), we evaluated whether the projected decreases in species density of each diel spectrum differed from those expected for random functional extinctions of the same number of species. Namely, we tested whether threatened species within diel niches share regions of trait space. We compared the potential decrease in spectrum species density considering the number of threatened species in each functional extinction scenario, with the mean loss of species density across 100 repetitions where the identity of threatened species was randomized within the diel niche. We found that, across nocturnal, cathemeral, and diurnal niches, the projected change in species density will be higher than expected in all extinction scenarios except the most conservative (Fig. 2A and table S3). The decrease in volume of the spectrum was greater than null expectations in all scenarios for nocturnal, crepuscular, and cathemeral species and for the VU and NT scenarios in diurnal mammals (Fig. 2B and table S3).

### Nocturnal global trait spectrum

Increased functional redundancy associated with greater taxonomic richness means that the nocturnal spectrum will experience the least change in density of any diel niche following functional extinctions (Figs. 1E and 2A). The functional loss of species within the Chiroptera hotspot will primarily occur in bats with more restricted habitat breadths, smaller body sizes, and larger litter sizes. However, should all threatened species be lost, the largest declines in species density are likely to occur among insectivorous bats, such as thick-eared bat (*Eptesicus pachyotis*), and small frugivorous arboreal foragers, such as Admiralty flying fox (*Pteropus admiralitatum*) and Moore’s woolly lemur (*Avahi mooreorum*) (15.4, 14.7, and 15.0% reduction in density, respectively, under the NT scenario).

### Crepuscular global trait spectrum

The decline of herbivores with a slow pace of life will result in a contraction in boundaries of the crepuscular trait spectrum associated with the functional extinction of medium-sized and large herbivores (Fig. 2B) coupled with the decline in density of trait combinations of up to 45.2% [e.g., red-fronted gazelle (*Eudorcas rufifrons*); Fig. 1F]. This will result in a shift in the density of occupation of nonthreatened species, with the formation of a new hotspot encompassing elephant shrews [*N* = 8; e.g., Western rock elephant shrew (*Elephantulus rupestris*); Fig. 1F].

### Cathemeral global trait spectrum

Following functional extinctions, there will be a shrinking in the boundaries of cathemeral trait space associated with the loss of megafauna [e.g., African buffalo (*Syncerus caffer*) and lion (*Panthera leo*)] and arboreal foragers (e.g., Lumholtz’s tree-kangaroo (*Dendrolagus lumholtzi*); Figs. 1C and 2B]. However, the largest shifts in species density will occur within the large cathemeral hotspot and will run parallel to the major trait gradients of body mass and diet (Fig. 1G). This will entail decreases in species density of up to 27.8% [e.g., Chinese shrew mole (*Uropsilus soricipes*)], occurring within species with limited habitat breadths and small litter sizes. Conversely, nonthreatened species within the hotspot tend to have broader habitat breadths and larger litter sizes.

### Diurnal global trait spectrum

Across scenarios and diel niches, diurnal mammals will experience the largest restructuring in the density of species occupation (Fig. 2A). Under the most extreme scenario, the decimation of diurnal primates will result in a decrease in species density of up to 84.8% [e.g., diademed sifaka (*Propithecus diadema*)] and the near disappearance of the first hotspot characterized by primates (Fig. 1H). This will result in a sizable shift in the relative density of species occupation along the fast-slow life history axis, with nonthreatened species being more likely to have a faster pace of life [e.g., Forrest’s rock squirrel (*Sciurotamias forresti*)]. In contrast, the clustering of threatened primates will lead to the smallest decrease in the volume of trait space of any diel niche (Fig. 2B).

### Biogeography of proportional declines in functional diversity across diel niches

Mammalian taxonomic richness and the distribution of activity patterns vary with the availability of biologically useful light (*33*), and threats to species are not distributed evenly across the world (*36*–*39*). Thus, the uneven and localized distribution of species in trait space might critically differentiate regional compared to global functional diversity, and the influence of individual functional extinctions might vary depending on the functional diversity of the local community.

**Fig. 3.**
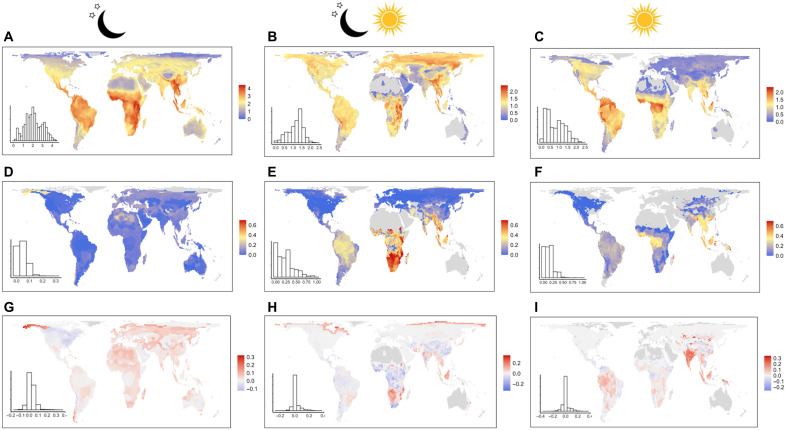
Biogeographic variation in lost functional diversity across diel niches following the NT functional extinction scenario. We show FD for (**A**) nocturnal (moon and stars), (**B**) cathemeral (moon, stars, and sun), and (**C**) diurnal (sun) species. (**D** to **F**) give the proportion of FD that is lost in each diel niche if all threatened species become functionally extinct under the NT functional extinction scenario (CR + EN + VU + NT + threatened DD). (**G** to **I**) Null expectations of whether the proportional functional loss in each diel niche is greater (i.e., functionally dispersed species are lost; red) or less (i.e., functionally redundant species are lost; blue) than expected if an equivalent number of species are lost at random. To avoid biasing the results from pixels with low species richness, within each diel niche, pixels with five species or fewer are excluded. Crepuscular richness is low over much of the land surface and does not allow meaningful analysis (but see fig. S3). Gray denotes where fewer than six species or no species are present. See fig. S3 for the proportional loss of FD under different functional extinction scenarios.

For all species, we downloaded IUCN range maps (*28*). On the basis of the same five functional traits used in the global analysis, thereby ensuring continuity across spatial scales, we calculated functional diversity for each diel niche as the total dendrogram branch lengths (termed FD; Fig. 3, A to C) (*42*, *43*). We then calculated the proportion of FD belonging to species that become functionally extinct under each scenario (Fig. 3, D to F). To avoid pixels with few species biasing the proportion analysis, we excluded those with fewer than six species in the diel niche of interest. This equated to almost all crepuscular pixels being excluded except in South America where most crepuscular species are not threatened; therefore, we did not include crepuscular mammals in this analysis (however, see fig. S3).

There was marked geographical variation across diel niches in FD, with nocturnal and diurnal FD being the greatest in the tropics (Fig. 3, A and B) and cathemeral FD being the highest in the upper latitudes, Southeast Asia, and East Africa (Fig. 3C). As the functional influence on ecosystems of threatened species is lost, there will be substantial variation across diel niches in the proportion of lost FD (Fig. 3, D to F). High taxonomic richness of nocturnal mammals, particularly across the tropics, results in a relatively lower proportion of nocturnal FD being lost than in other diel niches (Fig. 3D). The FD of species active either partly (cathemeral) or fully (diurnal) during the daytime will decline most severely across the tropics, notably in Southeast Asia where losses will take place among functionally dispersed species (Fig. 3, E and F). However, the greatest proportional declines in FD under the NT scenario will occur in cathemeral mammals in southern Africa associated with the loss of megaherbivores [across the land surface, this will lead to an upper interquartile (UIQ) loss of 28.0% compared to an UIQ loss of 23.1% in diurnal mammals; Figs. 2F and 3E and table S4].

### Biogeography of declining functional diversity relative to null expectations

We performed null models to estimate where proportional declines in diel FD are larger (i.e., higher functional dispersion) or smaller (i.e., higher functional redundancy) than expected, given the number of threatened species in each diel niche. We focused on the extreme extinction scenario where the functional influence of all threatened species will be lost (i.e., CR + EN + VU + NT + threatened DD). We compared the proportional loss of FD with 100 repetitions of the null model, where threatened species were randomly selected among all species present in that diel niche and pixel.

Across much of the land surface, nocturnal functional losses will occur in functionally dispersed species (Fig. 3G). In cathemeral mammals, losses will occur in functionally dispersed species in southern Africa associated with the functional extinction of megaherbivores weighing >1000 kg, in Southeast Asia related to the loss of threatened carnivores and medium-sized species, and in the higher northern latitudes due to the functional loss of polar bears (*Ursus maritimus*) (Fig. 3H). In contrast, across tropical Africa and the Americas, greater FD will be maintained than null expectations, because threatened species are clustered in trait space (Fig. 3H). In those mammals active primarily during the daytime, disproportionate losses will occur in functionally dispersed species, notably in India, Southeast Asia, and in the Amazon, as medium-sized species such as primates and carnivores are lost (Fig. 3I).

## DISCUSSION

We find an elevated risk of functional extinctions in diurnal mammals under all scenarios that we modeled relative to those occupying other diel niches, suggesting that being active at the same time as people leaves species more vulnerable to anthropogenic pressures that may be greater during the daytime [e.g., disturbance (*7*), harvesting (*8*), and warming daytime temperatures (*44*, *45*)]. The functional loss of all threatened diurnal mammals will lead to deep functional losses in global trait space, which will be exacerbated in local communities, as they occur in functionally dispersed species. Ecosystems are strongly partitioned between day and night (*46*), and, as such, the disproportionate loss of day-active mammals, particularly in the tropics, will likely have profound implications for ecosystems as mammals active at other times of day may be unable to take up their roles as effectively.

The impending collapse of the world’s large herbivores (*38*) and the defaunation of the world’s primates (*36*) will constrict boundaries and drive shifting densities of species occupation in trait space. Megaherbivores (>45 kg) (*47*) are particularly vulnerable to human pressures (*18*, *20*) regardless of when they are active, and their loss will universally decrease the volume of occupied trait space as these trait combinations go extinct. The least amount of trait space will be lost in diurnal mammals associated with three nonthreatened megaherbivores [waterbuck (*Kobus ellipsiprymnus*), roan antelope (*Hippotragus equinus*), and forest hog (*Hylochoerus meinertzhageni*)]; however, population trends of all three species are currently declining, suggesting that local extirpation may be occurring and that this region of trait space may become threatened in the future (*29*). In contrast, although most primates are vulnerable to functional extinctions (data file S2), the impact this would have on trait space is dependent on diel niche. While 72% of nocturnal primates are threatened, functional diversity is likely to be maintained owing to functional redundancy from nonthreatened small (e.g., fruit bats) and large (e.g., tree-kangaroos) nonprimate arboreal foragers. Thus, redundancy is a key factor in understanding the response to functional extinctions within trait space. Conversely, the decimation of 90% of cathemeral primates along with arboreal foragers such as tree-kangaroos and threatened sloths has the potential to severely restrict boundaries of trait space with the extinction of these trait combinations. Almost a third of diurnal mammals are primates (32%), and the loss of 67% of these will lead to an extraordinary decrease in species density in trait space, illustrating the clustering of threatened species. Although, globally, these ecological strategies are maintained because of high levels of redundancy among primates, large areas of trait space will be left vulnerable to further loss of functional richness should secondary extinctions occur (*48*, *49*).

Natural variation in light and temperature has driven geographical variation in the distribution of mammalian functional diversity across the daily cycle. Except at higher latitudes where cathemerality dominates, most mammalian functional diversity elsewhere occurs at night. Although high levels of functional redundancy buffer nocturnal functional diversity within the global trait spectrum, within local communities, there is less redundancy built into the system, leaving functional diversity more vulnerable to species losses. Across much of the land surface, this will be compounded by losses occurring in functionally dispersed species, as those with a slower pace of life are favored by bushmeat hunters (*50*) and tend to be more vulnerable to habitat loss (*18*). A consequence is that declines in functional diversity will be greater than null expectations. In contrast, small secretive species that characterize the nocturnal niche, such as rodents and many bats, may be shielded from some anthropogenic pressures. Considering that half of mammalian FD is uniquely nocturnal (*16*), the erosion of nocturnal functional diversity may have profound repercussions for ecosystems, because mammals active at other times of day have different trait combinations and may not be able to as effectively carry out lost functions and processes.

Strict activity at dawn and dusk is relatively rare, leaving crepuscular functional diversity vulnerable to species losses, as shown by the diel spectrum experiencing the second largest decrease in species density (after diurnal mammals) and the largest loss in volume. However, only a small proportion of mammalian FD is uniquely crepuscular [~1%; (*16*)]; therefore, the overall impact of functional extinctions on the influence of mammals on ecosystems may be low.

Cathemerality is prevalent in the upper latitudes, particularly in very small species, and, when combined with the remoteness of many populations from human settlements, these hold the greater part of cathemeral functional diversity. In contrast, within the tropics, activity during both the night and day is a common behavioral adaptation in megaherbivores (*51*) or carnivores and their prey (*52*). These species tend to be particularly vulnerable to anthropogenic pressures (*20*, *37*–*39*), and we show that a greater part of cathemeral functional diversity is threatened than of any other diel niche. Although herbivores weighing >1000 kg have vanished from nearly their entire prehistoric ranges (*53*), the disproportionate impact on functional diversity of the loss of those remaining species from their stronghold in southern Africa underscores what would have taken place globally. Conceivably, because cathemeral mammals are active both during the daytime and nighttime, the loss of their functional role from local communities could be taken up by diurnal or nocturnal species, respectively. However, being very small, very large, or carnivorous, many of these species fill important functional roles, the loss of which may disproportionately affect ecosystem functionality.

As the simulated functional extinctions progress through time from the near (CR scenario) to longer (NT scenario) term, diurnal mammals will consistently experience the deepest losses to functional diversity globally, and this is compounded locally by the disproportionate loss of functionally dispersed species. The decline of mammalian functional diversity during the daytime is being further exacerbated by an increasing trend of day-active mammals switching their activity to the nighttime as species strive to escape from anthropogenic pressures [e.g., human disturbance (*7*) and warming daytime temperatures (*44*, *45*)]. Currently, 69.5% of non-fossorial, terrestrial mammals are nocturnal: At the extreme, if all threatened species become functionally extinct and all those non-nocturnal species that can apparently switch their activity to the nighttime do so, then this will shift the distribution of mammalian functional diversity toward nocturnality with 78.3% of nonthreatened species being active at night. It is likely that a decline in the diurnal and relative increase in the nocturnal influence of mammals on ecosystems will have profound but currently unrealized impacts. However, the nighttime environment is also increasingly vulnerable to change; over much of the land surface, it is warming more quickly than the daytime (*6*), while the increasing prevalence of artificial light at night (*54*) and its use for bushmeat hunting (*55*) add further pressure on already stressed ecosystems.

Given the marked differences in environmental conditions and community structures between daytime and nighttime, it is almost inevitable that ecosystem functioning will also differ (*46*). Here, we show that the diel axis is a previously overlooked dimension to understanding the impact of functional losses in vertebrates. Species active at different times of day can either have unique roles that expand functional diversity (*16*) or they can replicate functional diversity occurring at other times of day, increasing ecosystem stability and resilience (*56*, *57*). However, there is currently limited understanding of how these processes work within local communities, across different ecosystems, or the ability of other taxa to take up these functions. Hopefully, new initiatives, such as temporal exclusion experiments, will inform on the ecosystem impacts of diel variation in species losses dependent on specific ecosystem functions, such as pollination (*24*) or predation (*58*). Furthermore, human pressures such as habitat loss, harvesting, or climate change also vary temporally and spatially, and the degree to which their impacts are additive or synergistic and how the balance between these pressures varies across the 24-hour cycle are unclear.

## MATERIALS AND METHODS

For 5033 extant terrestrial mammals, we obtained IUCN conservation status, current range distribution, and data on their diel niche and a further five functional traits (body mass, litter size, habitat breadth, foraging strata, and diet). We excluded sea mammals (*N* = 127, including two species of marine otter, *Enhydra lutris* and *Lontra felina*) and species described as highly or fully fossorial (*N* = 247), because these are likely to be reliant on different light cues than above-surface terrestrial species. All data processing and analyses were performed in R software for statistical computing v4.0.5 (*59*). Citations for R functions, packages, and package versions can be found in table S5.

### Functional extinction scenarios

We collected the conservation status of species from the IUCN Red list [version 2021-3, 2021 (*28*)] using the package “rredlist” (table S5). Species are classed as CR, EN, VU, NT, least concern, and DD based on population size reduction, geographic range, small population size and decline, very small or restricted population, or quantitative analysis (*28*). We used IUCN status here as a proxy for risk of functional extinction because both are associated with relative reductions in population size (*5*). A total of 649 species were classed as DD. To best assess the threat level for these species, we used published estimated classifications of threat status (*60*, *61*). These studies classed species as threatened (*N* = 297) or nonthreatened (*N* = 157) based on functional traits, rarity (geographical range size), and range loss (human encroachment on species range) together with phylogenetic and spatial dependencies (*60*, *61*). No information is available for the remaining 195 species, and so we took a conservative approach and considered these species to be nonthreatened.

To test how functional diversity within diel niches can be affected by species losses, following Toussaint *et al*. (*32*), we simulated the loss of threatened species in a progressive framework according to species IUCN status. CR species have the greatest risk of extinction and so were removed first. We then successively removed the species with lower categories of threat (CR + EN, CR + EN + VU, and CR + EN + VU + NT + threatened DD). The name of the scenario refers to the lowest IUCN threat category simulated as functionally extinct (e.g., VU scenario considers the functional extinction of all CR + EN + VU species). We focus on functional extinctions as opposed to actual extinctions, because these are more frequent and are more likely to be realized within a shorter time frame (*2*, *5*). These simulations then represent a gradient of risk of functional extinctions from a scenario where only the most endangered species go functionally extinct (most likely in the shorter term) to a more marked scenario where the functional influence of all threatened species (including NT and threatened DD species) is lost (could potentially occur in the medium to longer term).

### Functional traits

We extracted trait data from our recently compiled database for mammals [(*16*), which, in turn, sourced data from Cooke *et al*. (*34*), *Handbook of Mammals of the World* volumes 1 to 3 and 5 to 9 (*62*), PHYLACINE 1.2.1 (*63*), and EltonTraits 1.0 (*64*)] (data file S1). These traits reflect the spatiotemporal distribution of resource capture and release and can summarize species’ responses to environmental challenges (*34*, *65*). For full details on the compilation of these traits and the imputation of missing data, see Cox *et al*. (*16*). Updates to the activity patterns of 109 species can be found in data file S1.

In brief, following the *Handbook of Mammals of the World* (*62*), we assigned each species to one of four activity patterns: (i) nocturnal, active only at night (*N* = 3498); (ii) crepuscular, active only during twilight (around sunrise and sunset; *N* = 113); (iii) cathemeral, active throughout the day and night, interspersed with rest periods (*N* = 526); and (iv) diurnal, active only during the day (*N* = 896; data file S1). We collated information on a further five functional traits: body mass (mean adult body mass in grams), litter size (mean number of offspring per reproductive effort), diet (a continuous synthetic trait; see below), foraging strata (treated as ordinal; ground = 1, scansorial = 2, arboreal = 3, and aerial = 4) (*64*), and habitat breadth (number of IUCN habitats listed as suitable; data file S1). On the basis of the semi-quantitative records of 10 different diet categories, we calculated a continuous measure of a species’ diet. First, we calculated Gower distances between species based on diet data using the “gowdis” function in the FD package (table S5) before performing a principal components analysis (PCA) on the Gower distances using the “dudi.pco” function in the ade4 package (table S5). The first PC axis captured 40.7% of variation, and following Cooke *et al*. (*34*), only these values were used to serve as synthetic trait values [see Cox *et al*. (*16*)] for the validation of approach and analyses also including the second PC axis that captured an additional 18.7% of variation. Diet was predominantly loaded positively on invertebrates and vertebrates and negatively on plant material and seeds, thus representing a gradient from invertivore to herbivore, reflecting previous diet ordinations (*16*, *34*, *66*).

Trait data were not available for all species, but, overall, less than 5% of trait values were missing (activity patterns, 3%; body mass, 1%; litter size, 24%; foraging strata, 1%; and habitat breath, 0.1%). To achieve complete species-trait coverage, we imputed missing data using the Multivariate Imputation by Chained Equations package (table S5) based on the ecological (the transformed traits) and phylogenetic [the first 10 phylogenetic eigenvectors extracted from trees were obtained from the PHYLACINE 1.2.1 database (*67*)] relationships between species. We then randomly selected one of the 25 possible datasets extracted from data imputation (*16*) and used this for all analyses (dataset 24; data file S1). The reliability of the imputed data compared to a data deletion approach has been previously validated (*16*) and was also found to be robust here (see the “Sensitivity tests” section).

### Functional extinctions and diel niche

We tested whether the risk of functional extinctions varied across progressive extinction scenarios dependent on diel niche. Data for diel niche were phylogenetically imputed for 3% of species (nocturnal, *N* = 141; crepuscular, *N* = 1; cathemeral, *N* = 1; and diurnal, *N* = 12) (*14*). Inclusion of phylogenetically imputed traits in phylogenetic generalized linear models (GLMs) can cause issues of circularity (*67*). However, species with imputed data are biased toward nocturnality; therefore, to avoid weighting the analysis, we included species with imputed data and analyzed the data using generalized linear mixed models while including genus as a random effect. For each scenario, we regressed the binomial response of whether a species goes functionally extinct (1) or not (0) against the four-level factor diel niche. To test the sensitivity of our results, we repeated the analysis with a phylogenetic GLM (*68*) under the data deletion approach (nocturnal, *N* = 3,357; crepuscular, *N* = 112; cathemeral, *N* = 525; and diurnal, *N* = 883) (table S2 and data file S1).

### Shifting densities in diel global trait spectra

First, for each diel niche and following (*16*, *29*–*32*), we built a two-dimensional global trait spectrum from the transformed (log_10_ for body mass and litter size and square root for habitat breadth) and standardized traits via PCA using the “princomp” function in the vegan package (table S5). The ordination of species across this surface represents a two-dimensional continuum, integrating ecological strategies within each of the five trait dimensions. We used multivariate kernel density estimation to calculate the occurrence probability of given combinations of trait values (probability contours) across the trait spectra (*31*) for all species via the “kde” function in the ks package (table S5). In each case, we extracted contours at the 0.5, 0.95, and 0.99 quantiles of the probability distribution, thus highlighting the regions of the highest and lowest trait occurrence probability (Fig. 1, A to D). Because the results depend on the choice of bandwidth used for the smoothing kernel, we used unconstrained bandwidth selectors that were the sum of the asymptotic mean squared error pilot bandwidth selection (*69*) through the “Hpi” function in the ks package (table S5).

Second, under progressive functional extinction scenarios, we examined changes in density of species occupation within the trait spectrum for each diel niche if threatened species go functionally extinct and, thus, no longer contribute to mammalian FD and are therefore effectively extinct from trait space. For each scenario, we calculated kernel density estimates as above for the occurrence probability of trait combinations for the remaining species only. We then converted kernel density estimates for all species and for the remaining species only to raster objects, using the “raster” function in the raster package (table S5). Values were converted to a proportion by dividing each pixel by the maximum value in the raster, before calculating the difference between density estimates within the 0.99 quantile in diel trait spectra for all species and for nonthreatened species. The color gradient thus depicts regions where the relative density of trait combinations will decrease (red) or increase (blue) following functional extinctions. Thus, regions with a high loss of density depict clustering of threatened species in trait space (e.g., diurnal primates; Fig. 1H). This approach considers the differences in density within boundaries of trait space and informs on the differences between functional spectra, particularly in cases where functional redundancy is high within diel niches.

To quantify the degree to which functional diversity is lost in each diel niche under progressive extinction scenarios, we took two approaches. First, approximately following Toussaint *et al*. (*32*), we calculated the change in absolute density for each pixel as the absolute value of the relative change in density, averaged across pixels. Second, we determined the decrease in volume of each diel trait spectrum as the proportion of pixels within the full spectrum that fall outside of the spectrum boundaries following each functional extinction scenario.

To assess whether the impacts of the potential functional loss of species in each diel niche are different from what would be expected if the risk of functional extinction is not related to species’ traits, we compared the observed changes in the trait spectra to a null model where the functionally extinct species are randomly selected from within the diel niche. For each scenario, we compared the loss of functional diversity to 100 losses of functional diversity where the species traits of threatened species were randomly selected among the species from that diel niche. This allowed us to assess whether losing threatened species reduces more or less than expected the diel functional spectra.

### Biogeography of functional extinctions

Global trait spectra encompass all species in each diel niche; however, mammalian functional diversity shows marked geographic variation (*18*), and the distribution of activity patterns varies with the availability of biologically useful light (*33*). The uneven and localized distribution of species in trait space might critically differentiate functional diversity within small regions. Potential functional extinctions might thus cause marked erosion of functional diversity locally. Considering that species and their threats are not distributed evenly across the world (*4*, *70*), the impact of the functional loss of threatened species in specific regions might differ from the global pattern.

Maps of the current mammalian ranges were downloaded from IUCN version 2016-3 (www.iucnredlist.org/resources/spatial-data-download). Species polygons were filtered to include only those coded by the IUCN as follows: presence: “extant”; origin: “native,” “reintroduced,” or “origin uncertain”; and seasonality: “resident,” “breeding season,” “nonbreeding season,” or “seasonal occurrence uncertain.” We rasterized the polygon for each species and reprojected to Behrmann cylindrical equal area [EASE-Grid 2.0: EPSG:6933 (*71*)] at a resolution of 96.5 km by 96.5 km, where a pixel was assigned a value of 1 if it was overlapped by any of the polygon, thus incorporating species with small ranges (raster package; table S5). Otherwise, pixels were assigned a value of 0. IUCN range maps were unavailable for 13 species (nocturnal, 9; cathemeral, 1; and diurnal, 3). For these species, we used current range maps provided by PHYLACINE 1.2.1 at a resolution of 96.5 km by 96.5 km in Behrmann cylindrical equal area (*63*).

For all species, we created a presence-absence matrix, where columns were species and rows were pixels. For each diel niche and pixel, we (i) calculated functional diversity as the total branch lengths in a functional dendrogram [termed FD (*42*, *43*)], (ii) determined the proportion of FD predicted to be lost under progressive functional extinction scenarios, and (iii) identified regions where the loss in FD is predicted to be greater or lower than would be expected if an equivalent number of species to those threatened were lost by chance. FD provides a single index that encompasses how dispersed a set of species are in trait space, describes the functional relationships shared by species (*42*), accounts for clustering of species (functional redundancy), and has been widely used with presence/absence data such as those analyzed here. Once a dendrogram has been generated for an assemblage, species branches can be removed (“pruned”) without changing the relative positions of the remaining species. Thus, FD provides a highly flexible approach for calculating the contribution of subsets of species [i.e., (i) diel niche and (ii) nonthreatened species under progressive functional extinction scenarios] to the FD of the entire mammalian assemblage.

Following the existing practice (*72*, *73*), we calculated multivariate trait dissimilarity using Gower’s distance for each pairwise combination of all 5033 species in the dataset using the gowdis function in the FD package (table S5). Equal weight was given to each of the traits. The functional dendrogram was built using the “hclust” function with UPGMA (unweighted pair group method with arthmetric mean) clustering, which ensures the most faithful preservation of the original distances in the dissimilarity matrix (*74*). The “prune” function in the dendextend package allows the user to remove targeted branches of the dendrogram, without altering the lengths between the remaining branches (i.e., the distance between two species remains constant across multiple dendrograms; table S5). For each pixel and diel niche, the functional dendrogram was pruned of the branches of all species belonging to other diel niches and species of the diel niche that were not present in the pixel. FD was then calculated as the sum of lengths of connecting segments in the resulting dendrogram (*42*, *43*) using the “treeheight” function in the vegan package (table S5). Because using a dendrogram approach might, in some cases, artificially increase the functional distances between species that have similar trait values, we quantified the mean square deviation to assess the congruence between the functional distance as given by Gower’s metric and the cophenetic distance on the functional dendrogram (*75*, *76*). The mean absolute deviation equaled to 0.047, suggesting the average absolute deviation between Gower’s and the dendrogram-based distances of approximately 4.7%. The correlation between Gower’s distance matrix and the distance matrix based on the functional dendrogram equaled to 0.79, further indicating minimal loss of information.

For each functional extinction scenario and pixel, we repeated the above except for the fact that we also pruned species in the diel niche of interest that had become functionally extinct. We then calculated the proportion of FD in each pixel that belonged to nonthreatened species. When species richness is low, FD may be overly weighted by the contribution of single species. Therefore, to remove these potential sources of bias in the analysis, we excluded pixels that contained five species or fewer in that diel niches.

### Biogeographic variation in functional extinction in functionally dispersed and redundant species

For each diel niche, we assessed whether the impacts of potential functional extinctions among threatened species disproportionately occur among functionally dispersed or functionally redundant species. For each diel niche and pixel, we compared the observed changes in FD to a null model where functional extinctions occur randomly from among the diel species pool in the pixel. For the most extreme functional extinction scenario, we compared loss of FD to 100 losses of FD, where the traits of threatened species were randomly selected among the pixel pool of species. We then calculated the mean proportional loss in FD across 100 simulations and present the mean simulated less relative to the observed changes in FD if all threatened species are lost.

### Sensitivity tests

Overall, our results and conclusions were qualitatively similar under each functional extinction scenario with and without imputed trait data for (i) proportions of threatened species in each scenario [compare table S1 (i) with table S1 (ii)]; (ii) outputs from the GLMM models [compare table S2 (i) with table S2 (ii)]; (iii) trait spectra and change in density (compare fig. S1 with fig. S4); (iv) trait spectra for nonthreatened and threatened species (compare fig. S2 with fig. S5); (v) change in trait spectra absolute density and volume (compare Fig. 2 with fig. S6); and (vi) geographical variation in FD and proportional loss under progressive extinction scenarios (compare fig. S3 with fig. S7). We also tested the sensitivity of our results with respect to the identity of the traits for (vii) global patterns of diel FD (compare Fig. 3, A to C, with fig. S8) and (viii) lost FD under the NT functional extinction scenario (compare Fig. 3, D to F, with fig. S9).

## Supplementary Materials

### This PDF file includes:

Supplementary Text Figs. S1 to S9 Tables S1 to S5 References
