# Supplementary material for: Global and regional erosion of mammalian functional diversity across the diel cycle
Source: Sci Adv. 2022 Aug 12;8(31):eabn6008. doi: 10.1126/sciadv.abn6008 (PMC9374345; doi:10.1126/sciadv.abn6008)
Supplement: Supplementary file 1 — Supplementary Text Figs. S1 to S9 Tables S1 to S5 References [file sciadv.abn6008_sm.pdf]

Science Advances
AAAS

Supplementary Materials for

Global and regional erosion of mammalian functional diversity across the diel cycle

Daniel T. C. Cox *et al.*

Corresponding author: Daniel T. C. Cox, d.t.c.cox@exeter.ac.uk

Sci. Adv. **8**, eabn6008 (2022)
DOI: 10.1126/sciadv.abn6008

The PDF file includes:

Supplementary Text
Figs. S1 to S9
Tables S1 to S5
Legends for data S1 and S2
References

Other Supplementary Material for this manuscript includes the following:

Data S1 and S2

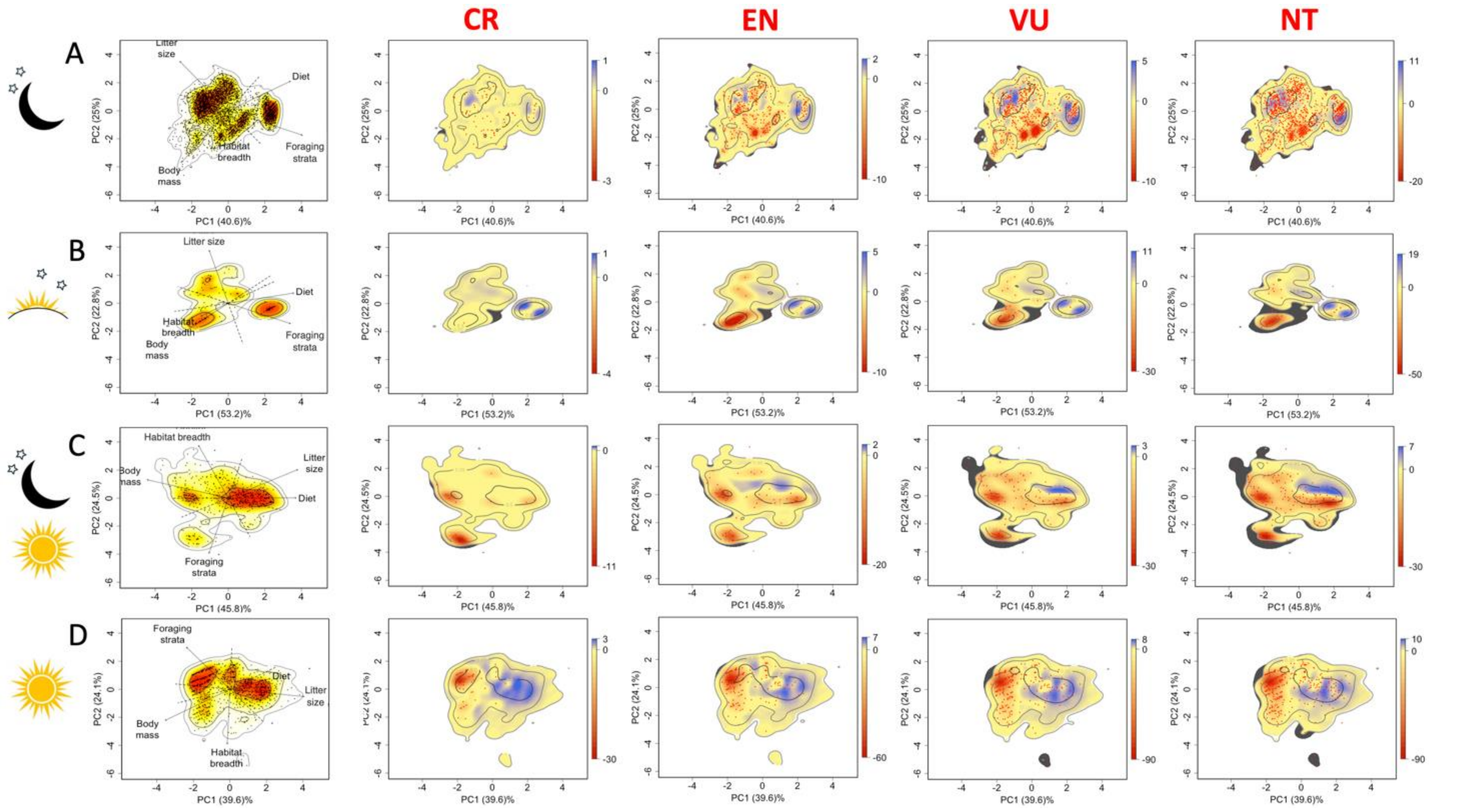

Fig. S1.

Erosion of diel trait spectra following progressive functional extinctions. For (**A**) nocturnal (moon and stars), (**B**) crepuscular (sunset/sunrise and stars), (**C**) cathemeral (moon, stars and sun), and (**D**) diurnal mammals (sun), we give the global trait spectra for

all species (far left panels). Projections show the species (dots) defined by principal component (PC) axes, and percentage values give the proportion of the total variation explained. Solid arrows indicate the direction and weighting of traits analyzed. The color gradient specifies regions of highest (red) to lowest (white) occurrence probability of species, with contour lines indicating 0.5, 0.95 and 0.99 quantiles. We then started by removing species with a higher risk of becoming functionally extinct (CR), then progressively removed additional species from categories with lower threat (EN, VU, NT). The title above the panel column refers to the lowest IUCN threat category from which all species in this and higher threat categories are simulated as functionally extinct. Panels depict shifts in density of species occupation as threatened species are progressively lost. Contour lines show quantiles for remaining species, and red dots are the position of threatened species. Red shading reflects areas where estimated density is lower following functional extinctions (i.e., those traits become relatively less frequent at the global scale) and blue shading shows where estimated density is higher after extinctions (i.e., those traits become relatively more frequent at the global scale). Grey areas are functional space within the 0.99 quantile for all species, but above the 0.99 quantile for remaining species (i.e., a reduction in volume of trait space).

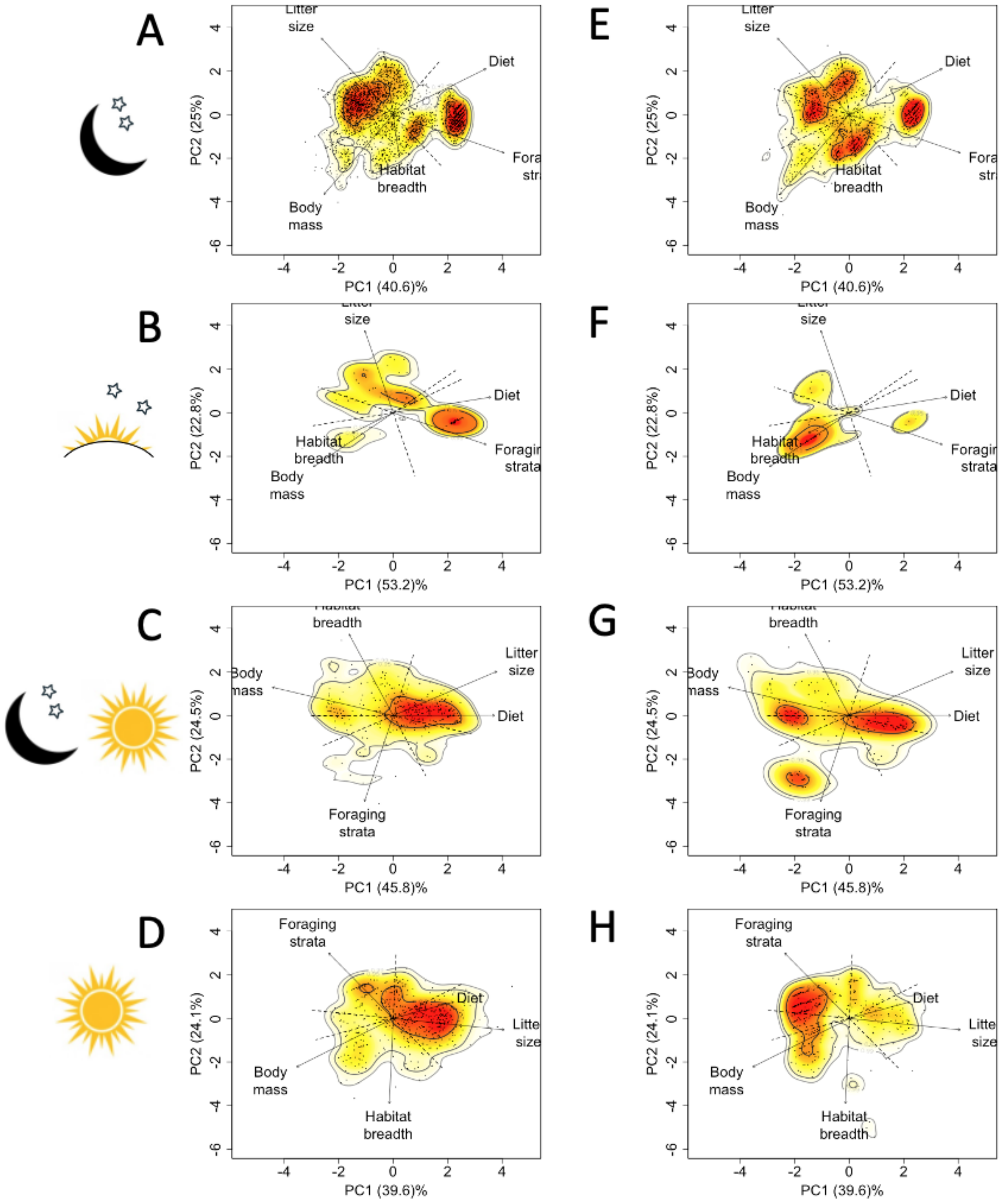

Fig. S2.

Diel global trait spectra for (A-D) non-threatened, and (E-F) threatened species (CR + EN + VU + NT + threatened DD). We show (A, E) nocturnal (*N* = 3,498), (B, F) crepuscular (*N* = 113), (C, G) cathemeral (*N* = 526), and (D, H) diurnal (*N* = 896) species.

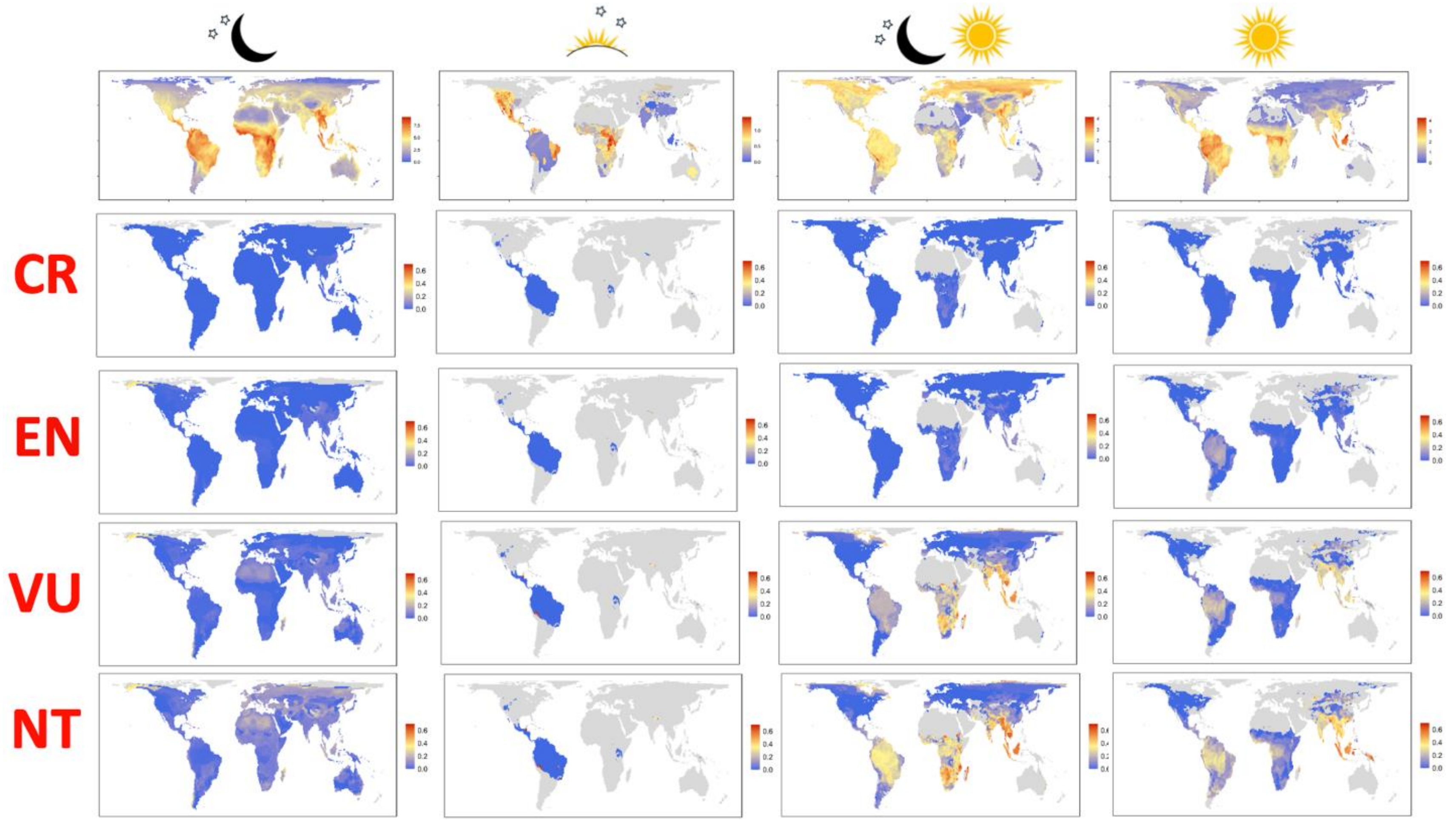

Fig. S3.

Proportional loss in FD in each diel niche across functional extinction scenarios. The top panel shows FD of nocturnal (moon and stars silhouette), crepuscular (sunrise/sunset image), cathemeral (moon, stars and sun image) and diurnal (sun image) mammals. The panels

below show the proportional loss in FD in each pixel in each diel niche under progressive functional extinction scenarios. We started by removing species with a higher risk of becoming functionally extinct (i.e., CR), then we continued progressively removing additional species from categories with lower threat. In the figures row titles refer to the lowest IUCN threat category simulated as functionally extinct.

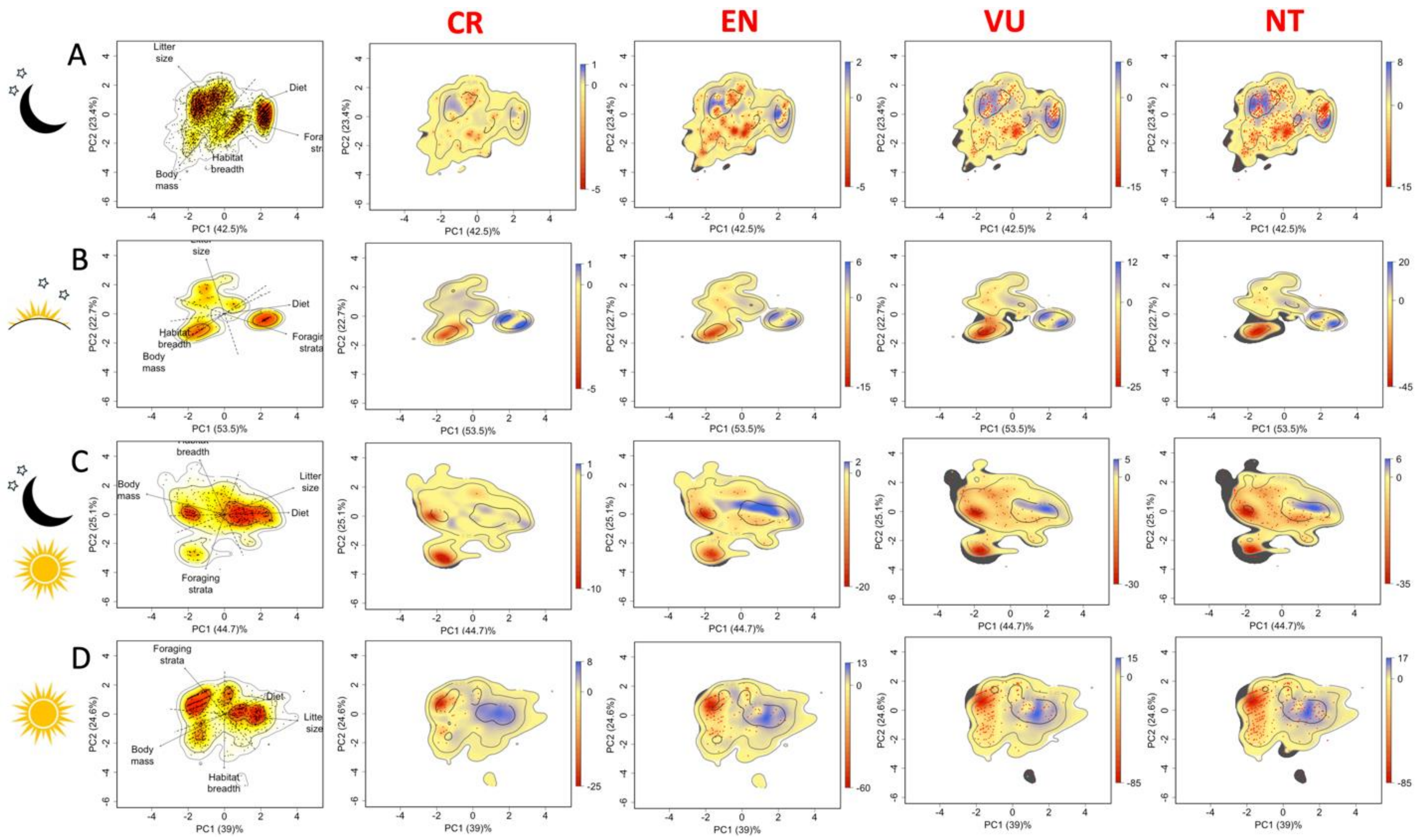

Fig. S4.

Erosion of diel trait spectra following progressive functional extinctions under the data deletion approach. For (**A**) nocturnal (moon and stars), (**B**) crepuscular (sunset/sunrise and stars), (**C**) cathemeral (moon, stars and sun), and (**D**) diurnal mammals (sun), we give

the global trait spectra for all species (far left panels). Projections show the species (dots) defined by principal component (PC) axes, and percentage values give the proportion of the total variation explained. Solid arrows indicate the direction and weighting of traits analyzed. The color gradient specifies regions of highest (red) to lowest (white) occurrence probability of species, with contour lines indicating 0.5, 0.95 and 0.99 quantiles. We then started by removing species with a higher risk of becoming functionally extinct (CR), then progressively removed additional species from categories with lower threat (EN, VU, NT). The title above the panel column refers to the lowest IUCN threat category from which all species in this and higher threat categories are simulated as functionally extinct. Panels depict shifts in density of species occupation as threatened species are progressively lost. Contour lines show quantiles for remaining species, and red dots are the position of threatened species. Red shading reflects areas where estimated density is lower following functional extinctions (i.e., those traits become relatively less frequent at the global scale) and blue shading shows where estimated density is higher after extinctions (i.e., those traits become relatively more frequent at the global scale). Grey areas are functional space within the 0.99 quantile for all species, but above the 0.99 quantile for remaining species (i.e., a reduction in volume of trait space).

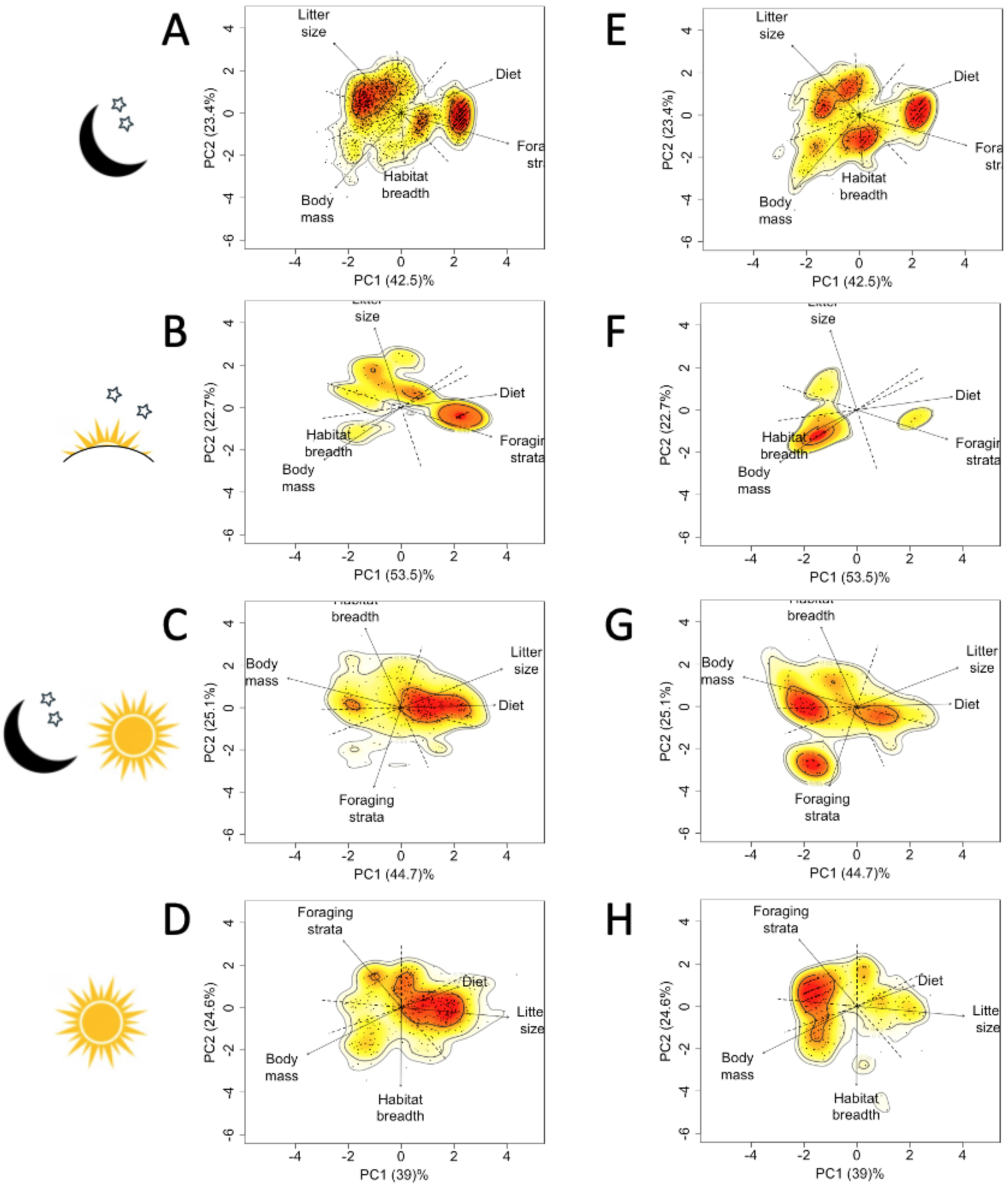

Fig. S5.

Diel trait spectra of (A-D) non-threatened, and (E-H) threatened (CR, EN, VU, NT and threatened DD) species under the data-deletion approach for traits. For nocturnal (moon and stars; N = 2,491), crepuscular (sunset/sunrise and star; N = 107), cathemeral (moon, stars and sun; N = 427), and diurnal mammals (sun; N = 753).

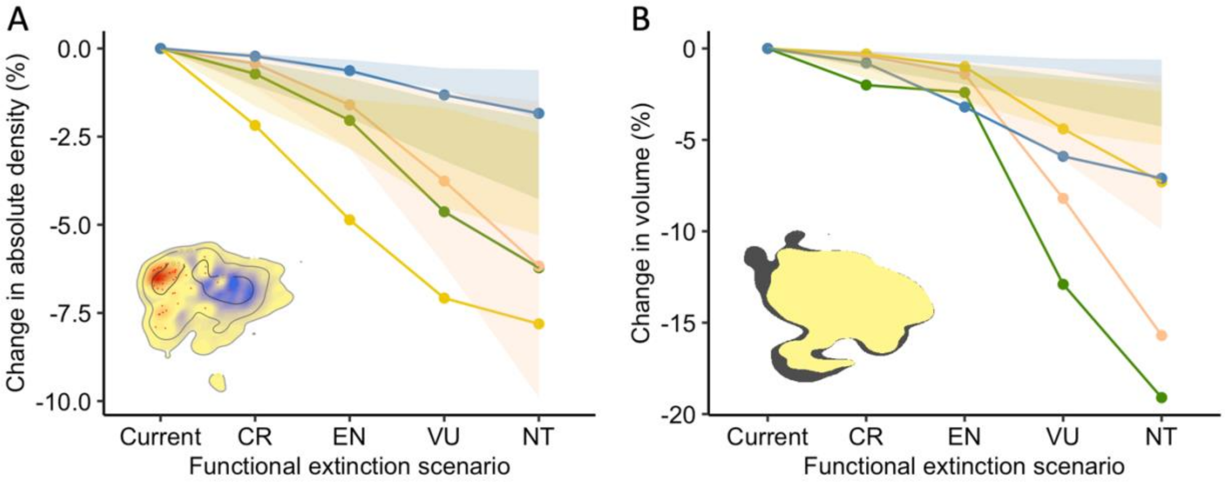

Fig. S6.

Loss in functional diversity in diel trait spectrum under data-deletion approach. Under the data deletion approach for each diel niche trait spectrum, we estimated the loss of functional diversity under progressive functional extinction scenarios as (**A**) the change in absolute density within the trait spectrum, and (**B**) the change in volume. Spectrum images on the panels illustrate examples of (**A**) change in density of species occupation, and (**B**) change in volume.

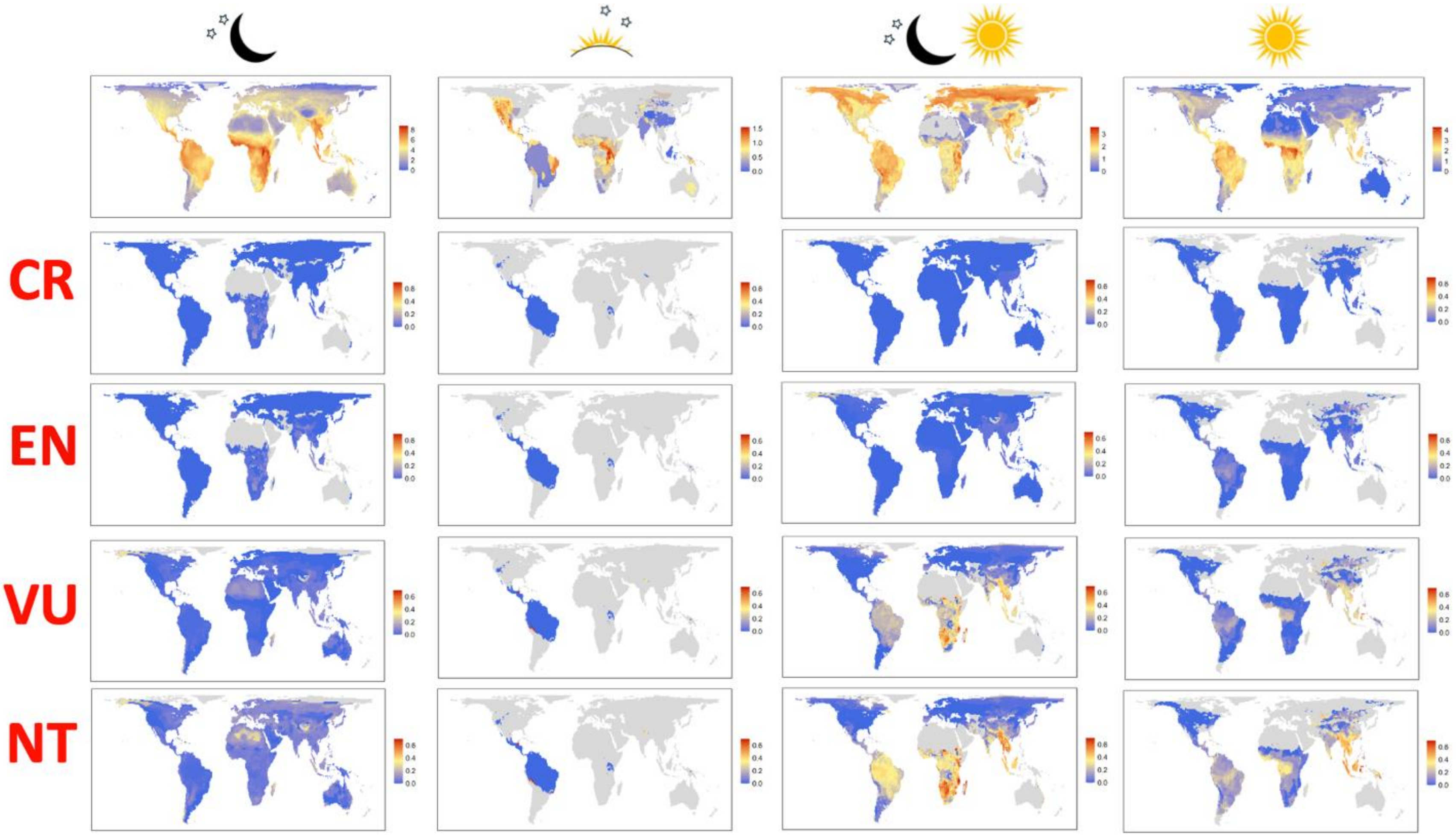

Fig. S7.

Under the data deletion approach, estimated biogeographic patterns of FD, and the proportional loss in FD under progressive functional extinction scenarios. The top panel shows FD of nocturnal (moon and stars silhouette), crepuscular (sunrise/sunset image), cathemeral (moon, stars and sun image) and diurnal (sun image) mammals. The panels below show the proportional loss in FD in each pixel in each diel niche under progressive functional extinction scenarios. We started by removing species with a higher risk of becoming functionally extinct (i.e., CR), then we continued progressively removing additional species from categories with lower threat. In the figures row titles refer to the lowest IUCN threat category simulated as functionally extinct.

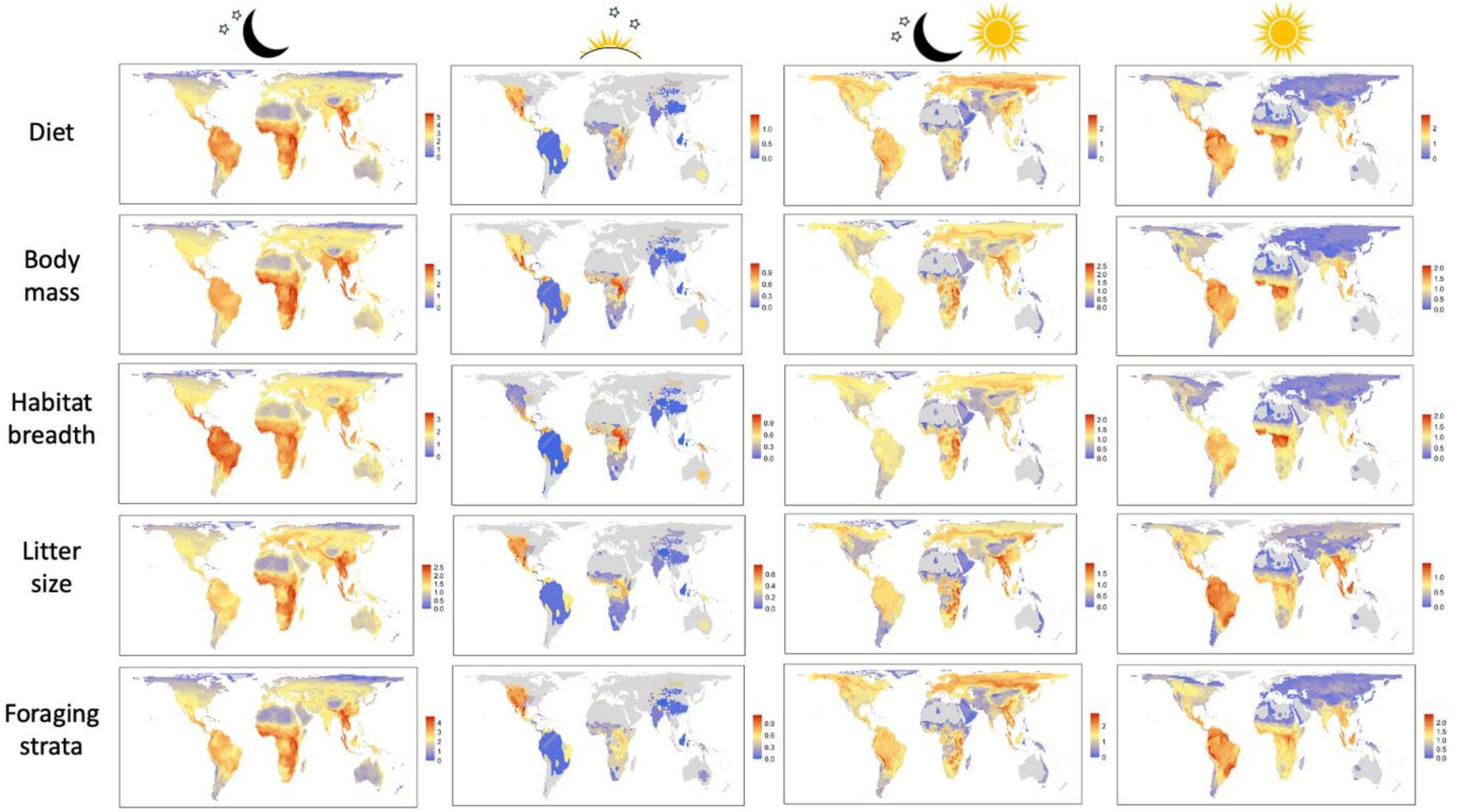

Fig. S8.

Biogeographic variation in FD in each diel niche (nocturnal (moon and stars silhouette), crepuscular (sunrise/sunset image), cathemeral (moon, stars and sun image) and diurnal (sun image) mammals), excluding each trait in turn. The trait name to the left of the panels gives the excluded trait.

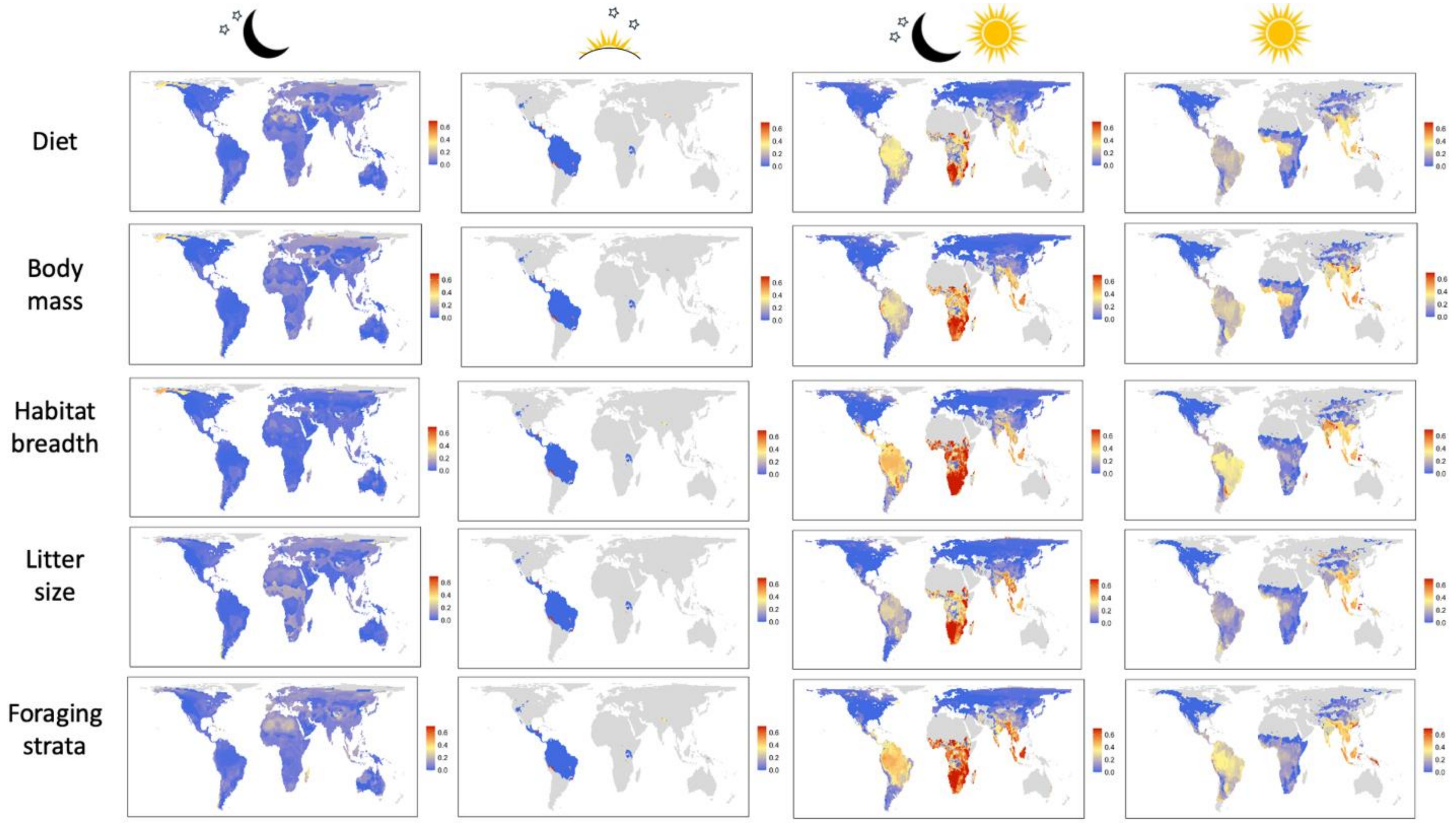

Fig. S9.

For each diel niche (nocturnal (moon and stars silhouette), crepuscular (sunrise/sunset image), cathemeral (moon, stars and sun image) and diurnal (sun image) mammals), the biogeographic variation in the proportion of lost FD under the NT functional extinction scenario, when excluding each functional trait in turn. The trait name to the left of the panels gives the excluded trait.

Table S1: The percentage of species in each diel niche that become functionally extinct under progressive functional extinction scenarios. (i) We simulated the loss of functional diversity by removing species based on their IUCN threat category in a progressive framework. We started by removing species with the higher risk of extinction (i.e., CR scenario), then we progressively removed additional species from categories with lower threaten status. In the table the name of the scenario refers to the lowest IUCN threat category simulated as functionally extinct. (ii) To test the sensitivity of our results with respect to imputed data, we also show the percentage of threatened species in each diel niche under the data deletion approach. Total species numbers are given in parentheses.

Scenario	Nocturnal	Crepuscular	Cathemeral	Diurnal
(i) N	(3,498)	(113)	(526)	(896)
CR	2.4% (84)	2.7% (3)	3.2% (17)	6.7% (60)
EN	10.2% (356)	10.6% (12)	10.6% (56)	19.6% (176)
VU	19.1% (669)	23.0% (26)	22.6% (119)	31.3% (280)
NT	31.6% (1,106)	34.8% (40)	34.7% (183)	41.3% (370)
(ii) Data deletion approach				
N	(2,491)	(107)	(427)	(753)
CR	1.9% (48)	1.9% (2)	3.0% (13)	6.8% (51)
EN	8.1% (201)	10.3% (11)	10.1% (43)	20.6% (155)
VU	16.5% (411)	23.4% (25)	23.0% (98)	32.3% (243)
NT	25.6% (637)	34.6% (37)	33.3% (142)	42.2% (318)

Table S2: GLMM results of a binary response of whether a species becomes functionally extinct under progressive functional extinction scenarios (1) or not (0), with diel niche. We started by removing species with a higher risk of becoming functionally extinct (i.e., CR scenario), then we continued progressively removing additional species from categories with lower threaten status. In the table the name of the scenario refers to the lower IUCN threat category simulated as functionally extinct. (i) To control for phylogeny, while avoiding issues of circularity resulting from using a phylogenetic tree to both impute missing trait data and in a phylogenetic GLM, we included Genus as a random effect (N = 133). We show coefficients relative to the base factor level of nocturnal, therefore a positive coefficient shows that species in that diel niche are more likely to go functionally extinct than nocturnal species. (ii) To test the sensitivity of our results with respect to imputed data, we repeated the analysis under the data-deletion approach (data S1) using a phylogenetic GLM (*68*). A phylogenetic tree was selected at random from PHYLACINE 1.2.1 (*63*). Standard errors are given in parentheses, * incidates a significant of <0.05, ** a significance of <0.01 and *** a significance of <0.001.

Scenario	Intercept				
	Nocturnal	Crepuscular	Cathemeral	Diurnal	
(i) N	3,498	113	526	896	*Random effect variance*
CR	-8.8 (±0.5)***	-0.9 (±1.3)	-0.3 (±0.6)	0.9 (±0.5)*	55.1 (±7.4)
EN	-3.0 (±0.2)***	-0.1 (±0.4)	0.002 (±0.2)	0.7 (0.2)***	3.2 (±1.8)
VU	-1.9 (±0.1)***	0.1 (±0.3)	0.2 (0.2)	0.7 (±0.2)***	2.6 (+1.6)
NT	-0.9 (±0.1)***	0.3 (±0.3)	0.2 (0.1)	0.5 (±0.1)***	1.6 (±1.3)
(ii) N	3,357	112	525	883	*Alpha*
CR	-3.5 (±0.2)***	0.7 (±0.4)	0.1 (±0.3)	1.1 (±0.2)***	0.12
EN	-2.1 (±0.1)***	0.1 (±0.3)	0.01 (±0.2)	0.9 (±0.1)***	0.19
VU	-1.6 (±0.1)***	0.7 (±0.2)**	0.6 (±0.1)***	0.8 (±0.1)***	0.17
NT	-0.9 (±0.05)***	0.7 (±0.2)***	0.7 (±0.1)***	0.8 (±0.1)***	0.25

Table S3: Change in diel trait spectra following progressive functional extinction scenarios. We started by removing species with a higher risk of becoming functionally extinct (i.e., CR scenario), then we continued progressively removing species from the categories with lower threatened risks. In the table the name of the scenario refers to the lower IUCN threat category simulated as functionally extinct. (i) Following functional extinctions, for each pixel in the spectrum we estimated the absolute change in relative density and averaged these values across pixels. (ii) The change in spectrum volume was calculated as the proportion of pixels within the full diel trait spectrum that fell outside of the spectrum boundaries following functional extinctions. Data underlying Fig. 2. To test the sensitivity of our results with respect to imputed data we repeated the analysis under the data deletion approach for (iii) the change in absolute density, and (iv) the change in volume of trait spectra. Data underlying fig. S6.

Scenario	Nocturnal	Crepuscular	Cathemeral	Diurnal
(i) Absolute change				
CR	0.046	0.12	0.70	1.16
EN	0.77	1.51	1.89	4.22
VU	1.19	3.53	4.11	6.60
NT	1.84	6.08	5.59	7.61
(ii) Volume change (%)				
CR	0.9	3.2	1.9	0.1
EN	4.0	4.0	2.74	0.1
VU	5.9	9.4	13.0	3.8
NT	7.2	19.9	18.3	6.28
Data deletion approach				
(iii) Absolute change				
CR	0.22	0.43	0.72	2.18
EN	0.63	1.60	2.04	4.86
VU	1.32	3.76	4.63	7.08
NT	1.84	6.17	6.22	7.81
(iv) Volume change (%)				
CR	0.8	0.4	2.0	0.3
EN	3.2	1.4	2.4	1.0

VU	5.9	8.2	12.9	4.4
NT	7.1	15.7	19.1	7.3

Table S4: Proportional geographical declines in mammalian diel FD under each functional extinction scenario. We present the median FD in each diel niche, and the proportional loss in FD under each functional extinction scenario. The lower and upper quantile ranges are given in parentheses. To avoid biasing the analysis through pixels with low numbers of species, those with five species or fewer were removed from the functional extinction scenario analyses.

Scenario	Nocturnal	Cathemeral	Diurnal
FD	2.1 (1.4; 2.8)	1.2 (0.9; 1.4)	0.8 (0.3; 1.2)
CR	0.0% (0.0%; 0.0%)	0.0% (0.0%; 0.0%)	0.0% (0.0%; 0.0%)
EN	0.0% (0.0%; 0.9%)	0.0% (0.0%; 0.0%)	0.0% (0.0%; 5.0%)
VU	1.6% (0%; 4.1%)	4.7% (0%; 19.2%)	2.7% (0%; 15.1%)
NT	6.6% (3.2%; 12.0%)	9.5% (1.1%; 28.0%)	12% (2.5%; 23.1%)

Table S5: R Functions, packages and package versions used in the analysis and cited in the main text.

Function	Package; version	Citation
rl_measures()	rredlist; 0.7.0	(*77*)
gowdis(); hclust()	FD; 1.0-12	(78)
dudi-pco()	ade4; 1.7-18	(79)
mice()	MICE; 3.13.0	(80)
kde(); Hpi()	ks; 1.11.7	(81)
prune()	dendextend; 1.14.0	(82)
princomp(); treeheight()	vegan; 2.5-6	(83)
raster(); mask(); projectRaster(); resample(); crop(); rasterise()	raster; 3.4.10	(84)

Data S1. (Separate file)

Trait data containing both missing values and imputed data, with data sources. Activity patterns we extracted from our recently compiled database for mammals (*16*). Here, we updated activity patterns for 109 species and provide data sources. We also now consider that the African forest elephant (*Loxodonta cyclotis*) is a separate species to the African savannah elephant (*Loxodonta africana*).

Data S2. (Separate file)

Taxonomic composition of non-threatened and threatened species under each functional extinction scenario. We started by removing species with a higher risk of becoming functionally extinct (i.e., CR), then we continued progressively removing species from the lower categories of threat. In the data frame the name of the scenario refers to the lower IUCN threat category simulated as functionally extinct.

REFERENCES AND NOTES

1. A. D. Barnosky, N. Matzke, S. Tomiya, G. O. U. Wogan, B. Swartz, T. B. Quental, C. Marshall, J. L. McGuire, E. L. Lindsey, K. C. Maguire, B. Mersey, E. A. Ferrer, Has the Earth's sixth mass extinction already arrived? *Nature* **471**, 51–57 (2011).

2. G. Ceballos, P. R. Ehrlich, R. Dirzo, Biological annihilation via the ongoing sixth mass extinction signaled by vertebrate population losses and declines. *Proc. Natl. Acad. Sci. U.S.A.* **114**, E6089–E6096 (2017).

3. D. Tilman, M. Clark, D. R. Williams, K. Kimmel, S. Polasky, C. Packer, Future threats to biodiversity and pathways to their prevention. *Nature* **546**, 73–81 (2017).

4. S. L. Pimm, C. N. Jenkins, R. Abell, T. M. Brooks, J. L. Gittleman, L. N. Joppa, P. H. Raven, C. M. Roberts, J. O. Sexton, The biodiversity of species and their rates of extinction, distribution, and protection. *Science* **344** (2014).

5. T. Säterberg, S. Sellman, B. Ebenman, High frequency of functional extinctions in ecological networks. *Nature* **499**, 468–470 (2013).

6. D. T. C. Cox, I. M. D. Maclean, A. S. Gardner, K. J. Gaston, Global variation in diurnal asymmetry in temperature, cloud cover, specific humidity and precipitation and its association with leaf area index. *Glob. Change Biol.* **26**, 7099–7111 (2020).

7. K. M. Gaynor, C. E. Hojnowski, N. H. Carter, J. S. Brashares, The influence of human disturbance on wildlife nocturnality. *Science* **360**, 1232–1235 (2018).

8. C. A. Peres, Synergistic effects of subsistence hunting and habitat fragmentation on Amazonian forest vertebrates. *Conserv. Biol.* **15**, 1490–1505 (2001).

9. D. Sanders, E. Frago, R. Kehoe, C. Patterson, K. J. Gaston, A meta-analysis of biological impacts of artificial light at night. *Nat. Ecol. Evol.* **5**, 74–81 (2021).

10. S. Peng, S. Piao, P. Ciais, R. B. Myneni, A. Chen, F. Chevallier, A. J. Dolman, I. A. Janssens, J. Peñuelas, G. Zhang, S. Vicca, S. Wan, S. Wang, H. Zeng, Asymmetric effects of daytime and night-time warming on Northern Hemisphere vegetation. *Nature* **501**, 88–92 (2013).

11. M. Galetti, R. Dirzo, Ecological and evolutionary consequences of living in a defaunated world. *Biol. Conserv.* **163**, 1–6 (2013).

12. R. Dirzo, H. S. Young, M. Galetti, G. Ceballos, N. J. B. Isaac, B. Collen, Defaunation in the Anthropocene. *Science* **345**, 401–406 (2014).

13. K. J. Gaston, J. P. Duffy, S. Gaston, J. Bennie, T. W. Davies, Human alteration of natural light cycles: Causes and ecological consequences. *Oecologia* **176**, 917–931 (2014).

14. B. W. Brook, N. S. Sodhi, C. J. A. Bradshaw, Synergies among extinction drivers under global change. *Trends Ecol. Evol.* **23**, 453–460 (2008).

15. C. R. Miller, B. T. Barton, L. Zhu, V. C. Radeloff, K. M. Oliver, J. P. Harmon, A. R. Ives, Combined effects of night warming and light pollution on predator-prey interactions. *Proc. R. Soc. B.* **284**, 20171195 (2017).

16. D. T. C. Cox, A. S. Gardner, K. J. Gaston, Diel niche variation in mammals associated with expanded trait space. *Nat. Commun.* **12**, 1753 (2021).

17. E. R. Pianka, L. J. Vitt, N. Pelegrin, D. B. Fitzgerald, K. O. Winemiller, Toward a periodic table of niches, or exploring the lizard niche hypervolume. *Am. Nat.* **190**, 601–616 (2017).

18. J. F. Brodie, S. Williams, B. Garner, The decline of mammal functional and evolutionary diversity worldwide. *Proc. Natl. Acad. Sci. U.S.A.* **118**, e1921849118 (2021).

19. F. Chichorro, A. Juslén, P. Cardoso, A review of the relation between species traits and extinction risk. *Biol. Conserv.* **237**, 220–229 (2019).

20. W. J. Ripple, C. Wolf, T. M. Newsome, M. G. Betts, G. Ceballos, F. Courchamp, M. W. Hayward, B. Van Valkenburgh, A. D. Wallach, B. Worm, Are we eating the world's megafauna to extinction? *Conserv. Lett.* **12**, e12627 (2019).

21. V. C. Foster, P. Sarmento, R. Sollmann, N. Tôrres, A. T. A. Jácomo, N. Negrões, C. Fonseca, L. Silveira, Jaguar and Puma activity patterns and predator-prey interactions in four Brazilian biomes. *Biotropica* **45**, 373–379 (2013).

22. M. S. McMunn, The timing of leaf damage affects future herbivory in mountain sagebrush (*Artemisia tridentata*). *Ecology* **98**, 1996–2002 (2017).

23. K. Wehner, L. Schäfer, N. Blüthgen, K. Mody, Seed type, habitat and time of day influence post-dispersal seed removal in temperate ecosystems. *PeerJ.* **8**, e8769 (2020).

24. C. J. Tremlett, M. Moore, M. A. Chapman, V. Zamora-Gutierrez, K. S.-H. Peh, Pollination by bats enhances both quality and yield of a major cash crop in Mexico. *J. Appl. Ecol.* **57**, 450–459 (2020).

25. Y. Tsunamoto, S. Naoe, T. Masaki, Y. Isagi, Different contributions of birds and mammals to seed dispersal of a fleshy-fruited tree. *Basic Appl. Ecol.* **43**, 66–75 (2020).

26. D. Mouillot, D. R. Bellwood, C. Baraloto, J. Chave, R. Galzin, M. Harmelin-Vivien, M. Kulbicki, S. Lavergne, S. Lavorel, N. Mouquet, C. E. T. Paine, J. Renaud, W. Thuiller, Rare species support vulnerable functions in high-diversity ecosystems. *PLoS Biol.* **11**, e1001569 (2013).

27. C. Violle, W. Thuiller, N. Mouquet, F. Munoz, N. J. B. Kraft, M. W. Cadotte, S. W. Livingstone, D. Mouillot, Functional rarity: The ecology of outliers. *Trends Ecol. Evol.* **32**, 356–367 (2017).

28. IUCN, The IUCN Red List of threatened species, version 2021-3 (2021); www.iucnredlist.org [accessed 21 March 2022].

29. C. P. Carmona, R. Tamme, M. Pärtel, F. de Bello, S. Brosse, P. Capdevila, R. González-M, M. González-Suárez, R. Salguero-Gómez, M. Vásquez-Valderrama, A. Toussaint, Erosion of global functional diversity across the tree of life. *Sci. Adv.* **7**, eabf2675 (2021).

30. R. S. C. Cooke, F. Eigenbrod, A. E. Bates, Projected losses of global mammal and bird ecological strategies. *Nat. Commun.* **10**, 2279 (2019).

31. S. Díaz, J. Kattge, J. H. C. Cornelissen, I. J. Wright, S. Lavorel, S. Dray, B. Reu, M. Kleyer, C. Wirth, I. C. Prentice, E. Garnier, G. Bönisch, M. Westoby, H. Poorter, P. B. Reich, A. T. Moles, J. Dickie, A. N. Gillison, A. E. Zanne, J. Chave, S. J. Wright, S. N. Sheremet'ev, H. Jactel, C. Baraloto, B. Cerabolini, S. Pierce, B. Shipley, D. Kirkup, F. Casanoves, J. S. Joswig, A. Günther, V. Falczuk, N. Rüger, M. D. Mahecha, L. D. Gorné, The global spectrum of plant form and function. *Nature* **529**, 167–171 (2016).

32. A. Toussaint, S. Brosse, C. G. Bueno, M. Pärtel, R. Tamme, C. P. Carmona, Extinction of threatened vertebrates will lead to idiosyncratic changes in functional diversity across the world. *Nat. Commun.* **12**, 5162 (2021).

33. J. J. Bennie, J. P. Duffy, R. Inger, K. J. Gaston, Biogeography of time partitioning in mammals. *Proc. Natl. Acad. Sci. U.S.A.* **111**, 13727–13732 (2014).

34. R. S. C. Cooke, A. E. Bates, F. Eigenbrod, Global trade-offs of functional redundancy and functional dispersion for birds and mammals. *Glob. Ecol. Biogeogr.* **28**, 484–495 (2019).

35. T. E. Lacher Jr., A. D. Davidson, T. H. Fleming, E. P. Gómez-Ruiz, G. F. McCracken, N. Owen-Smith, C. A. Peres, S. B. Vander Wall, The functional roles of mammals in ecosystems. *J. Mammal.* **100**, 942–964 (2019).

36. A. Estrada, P. A. Garber, A. B. Rylands, C. Roos, E. Fernandez-Duque, A. D. Fiore, K. A.-I. Nekaris, V. Nijman, E. W. Heymann, J. E. Lambert, F. Rovero, C. Barelli, J. M. Setchell, T. R. Gillespie, R. A. Mittermeier, L. V. Arregoitia, M. de Guinea, S. Gouveia, R. Dobrovolski, S. Shanee, N. Shanee, S. A. Boyle, A. Fuentes, K. C. MacKinnon, K. R. Amato, A. L. S. Meyer, S. Wich, R. W. Sussman, R. Pan, I. Kone, B. Li, Impending extinction crisis of the world's primates: Why primates matter. *Sci. Adv.* **3**, e1600946 (2017).

37. W. J. Ripple, J. A. Estes, R. L. Beschta, C. C. Wilmers, E. G. Ritchie, M. Hebblewhite, J. Berger, B. Elmhagen, M. Letnic, M. P. Nelson, O. J. Schmitz, D. W. Smith, A. D. Wallach, A. J. Wirsing, Status and ecological effects of the world's largest carnivores. *Science* **343**, e124148 (2014).

38. W. J. Ripple, T. M. Newsome, C. Wolf, R. Dirzo, K. T. Everatt, M. Galetti, M. W. Hayward, G. I. H. Kerley, T. Levi, P. A. Lindsey, D. W. Macdonald, Y. Malhi, L. E. Painter, C. J. Sandom, J. Terborgh, B. V. Valkenburgh, Collapse of the world's largest herbivores. *Sci. Adv.* **1**, e1400103 (2015).

39. W. J. Ripple, K. Abernethy, M. G. Betts, G. Chapron, R. Dirzo, M. Galetti, T. Levi, P. A. Lindsey, D. W. Macdonald, B. Machovina, T. M. Newsome, C. A. Peres, A. D. Wallach, C. Wolf, H. Young, Bushmeat hunting and extinction risk to the world's mammals. *R. Soc. Open Sci.* **3**, 160498 (2016).

40. C. P. Carmona, F. de Bello, N. W. H. Mason, J. Lepš, Trait probability density (TPD): Measuring functional diversity across scales based on TPD with R. *Ecology* **100**, e02876 (2019).

41. S. Villéger, N. W. H. Mason, D. Mouillot, New multidimensional functional diversity indices for a multifaceted framework in functional ecology. *Ecology* **89**, 2290–2301 (2008).

42. O. L. Petchey, K. J. Gaston, Functional diversity (FD), species richness and community composition. *Ecol. Lett.* **5**, 402–411 (2002).

43. O. L. Petchey, K. J. Gaston, Functional diversity: Back to basics and looking forward. *Ecol. Lett.* **9**, 741–758 (2006).

44. O. Levy, T. Dayan, W. P. Porter, N. Kronfeld-Schor, Time and ecological resilience: Can diurnal animals compensate for climate change by shifting to nocturnal activity? *Ecol. Monogr.* **89**, e01334 (2019).

45. T. C. Bonebrake, E. L. Rezende, F. Bozinovic, Climate change and thermoregulatory consequences of activity time in mammals. *Am. Nat.* **196**, 45–56 (2020).

46. K. J. Gaston, Nighttime ecology: The "nocturnal problem" revisited. *Am. Nat.* **193**, 481–502 (2019).

47. P. S. Martin, in *Quaternary Extinctions: A Prehistoric Revolution* (University of Arizona Press, 1989), pp. 354–403.

48. I. Donohue, O. L. Petchey, S. Kéfi, A. Génin, A. L. Jackson, Q. Yang, N. E. O'Connor, Loss of predator species, not intermediate consumers, triggers rapid and dramatic extinction cascades. *Glob. Change Biol.* **23**, 2962–2972 (2017).

49. A. Eklöf, B. Ebenman, Species loss and secondary extinctions in simple and complex model communities. *J. Anim. Ecol.* **75**, 239–246 (2006).

50. W. M. S. Souto, R. N. Lima, B. F. C. F. Sousa, Illegal bushmeat hunting and trade dynamics in a major road-hub region of the Brazilian Mid North. *Indian J. Tradit. Knowl.* **18**, 402–411 (2019).

51. T. Ramesh, R. Kalle, K. Sankar, Q. Qureshi, Role of body size in activity budgets of mammals in the Western Ghats of India. *J. Trop. Ecol.* **31**, 315–323 (2015).

52. Y. Wu, H. Wang, H. Wang, J. Feng, Arms race of temporal partitioning between carnivorous and herbivorous mammals. *Sci. Rep.* **8**, 1713 (2018).

53. Y. Malhi, C. E. Doughty, M. Galetti, F. A. Smith, J.-C. Svenning, J. W. Terborgh, Megafauna and ecosystem function from the Pleistocene to the Anthropocene. *Proc. Natl. Acad. Sci. U.S.A.* **113**, 838–846 (2016).

54. C. C. M. Kyba, T. Kuester, A. S. de Miguel, K. Baugh, A. Jechow, F. Hölker, J. Bennie, C. D. Elvidge, K. J. Gaston, L. Guanter, Artificially lit surface of Earth at night increasing in radiance and extent. *Sci. Adv.* **3**, e1701528 (2017).

55. M. Bowler, C. Beirne, M. W. Tobler, M. Anderson, A. DiPaola, J. E. Fa, M. P. Gilmore, L. P. Lemos, P. Mayor, A. Meier, G. M. Menie, D. Meza, D. Moreno-Gutierrez, J. R. Poulsen, A. de Souza Jesus, J. Valsecchi, H. R. El Bizri, LED flashlight technology facilitates wild meat extraction across the tropics. *Front. Ecol. Environ.* **18**, 489–495 (2020).

56. M. Houadria, N. Blüthgen, A. Salas-Lopez, M.-I. Schmitt, J. Arndt, E. Schneider, J. Orivel, F. Menzel, The relation between circadian asynchrony, functional redundancy, and trophic performance in tropical ant communities. *Ecology* **97**, 225–235 (2016).

57. A. Mougi, Diversity of biological rhythm and food web stability. *Biol. Lett.* **17**, 20200673 (2021).

58. C. J. Tambling, L. Minnie, J. Meyer, E. W. Freeman, R. M. Santymire, J. Adendorff, G. I. H. Kerley, Temporal shifts in activity of prey following large predator reintroductions. *Behav. Ecol. Sociobiol.* **69**, 1153–1161 (2015).

59. R Development Core Team, R: A language and environment for statistical computing (2019); http://R-project.org.

60. L. M. Bland, B. Collen, C. D. L. Orme, J. Bielby, Predicting the conservation status of data-deficient species. *Conserv. Biol.* **29**, 250–259 (2015).

61. W. Jetz, R. P. Freckleton, Towards a general framework for predicting threat status of data-deficient species from phylogenetic, spatial and environmental information. *Philos. Trans. R. Soc. Lond. B Biol. Sci.* **370**, 20140016 (2015).

62. R. Mittermeier, A. Rylands, T. Lacher, D. Wilson, in *Handbook of the Mammals of the World* (Lynx Edicions, 2001), vols. 1–3 and 5–9.

63. S. Faurby, M. Davis, R. Ø. Pedersen, S. D. Schowanek, A. Antonelli, J.-C. Svenning, PHYLACINE 1.2: The phylogenetic atlas of mammal macroecology. *Ecology* **99**, 2626 (2018).

64. H. Wilman, J. Belmaker, J. Simpson, C. de la Rosa, M. M. Rivadeneira, W. Jetz, EltonTraits 1.0: Species-level foraging attributes of the world's birds and mammals. *Ecology* **95**, 2027 (2014).

65. D. F. B. Flynn, M. Gogol-Prokurat, T. Nogeire, N. Molinari, B. T. Richers, B. B. Lin, N. Simpson, M. M. Mayfield, F. DeClerck, Loss of functional diversity under land use intensification across multiple taxa. *Ecol. Lett.* **12**, 22–33 (2009).

66. S. Pineda-Munoz, J. Alroy, Dietary characterization of terrestrial mammals. *Proc. R. Soc. B Biol. Sci.* **281**, 20141173 (2014).

67. C. Penone, A. D. Davidson, K. T. Shoemaker, M. D. Marco, C. Rondinini, T. M. Brooks, B. E. Young, C. H. Graham, G. C. Costa, Imputation of missing data in life-history trait datasets: Which approach performs the best? *Methods Ecol. Evol.* **5**, 961–970 (2014).

68. A. R. Ives, T. Garland Jr., Phylogenetic logistic regression for binary dependent variables. *Syst. Biol.* **59**, 9–26 (2010).

69. T. Duong, M. Hazelton, Plug-in bandwidth matrices for bivariate kernel density estimation. *J. Nonparametr. Stat.* **15**, 17–30 (2003).

70. C. Howard, C. H. Flather, P. A. Stephens, A global assessment of the drivers of threatened terrestrial species richness. *Nat. Commun.* **11**, 993 (2020).

71. M. J. Brodzik, B. Billingsley, T. Haran, B. Raup, M. H. Savoie, EASE-Grid 2.0: Incremental but significant improvements for earth-gridded data sets. *ISPRS Int. J. Geo-Inf.* **1**, 32–45 (2012).

72. M. A. Jarzyna, W. Jetz, A near half-century of temporal change in different facets of avian diversity. *Glob. Change Biol.* **23**, 2999–3011 (2017).

73. S. Pavoine, J. Vallet, A.-B. Dufour, S. Gachet, H. Daniel, On the challenge of treating various types of variables: Application for improving the measurement of functional diversity. *Oikos* **118**, 391–402 (2009).

74. B. Mérigot, J.-P. Durbec, J.-C. Gaertner, On goodness-of-fit measure for dendrogram-based analyses. *Ecology* **91**, 1850–1859 (2010).

75. E. Maire, G. Grenouillet, S. Brosse, S. Villéger, How many dimensions are needed to accurately assess functional diversity? A pragmatic approach for assessing the quality of functional spaces. *Glob. Ecol. Biogeogr.* **24**, 728–740 (2015).

76. S. Villéger, E. Maire, F. Leprieur, On the risks of using dendrograms to measure functional diversity and multidimensional spaces to measure phylogenetic diversity: A comment on Sobral *et al.* (2016). *Ecol. Lett.* **20**, 554–557 (2017).

77. S. Chamberlain, rredlist: IUCN Red List (2020); https://CRAN.R-project.org/package=rredlist.

78. E. Laliberté, P. Legendre, B. Shipley, FD: Measuring functional diversity from multiple traits, and other tools for functional ecology, R package version 1.0-12.1 (2014); https://CRAN.R-project.org/package=FD.

79. S. Dray, A.-B. Dufour, The "ade4" package: Implementing the duality diagram for ecologists. *J. Stat. Softw.* **22**, 1–20 (2007).

80. S. van Buuren, MICE: Multivariate imputation by chained equations in R. *J. Stat. Softw.* **453**, 1–67 (2011).

81. T. Duong, ks: Kernel smoothing, R package version 1.11-7 (2020); https://CRAN.R-project.org/package=ks.

82. T. Galili, dendextend: An R package for visualizing, adjusting, and comparing trees if hierarchical clustering. *Bioinformatics* **31**, 3718–3720 (2015).

83. J. Oksanen, F. G. Blanchet, M. Friendly, R. Kindt, P. Legendre, D. McGlinn, P. R. Minchin, R. B. O'Hara, G. L. Simpson, P. Solymos, M. H. H. Stevens, E. Szoecs, H. Wagner, vegan: Community ecology package (2019); https://CRAN.R-project.org/package=vegan.

84. R. J. Hijmans, raster: Geographic data analysis and modeling, R package version 3.4-10 (2021); https://CRAN.R-project.org/package=raster.
